# Supplementary material for: Modeling the Impact of Exogenous Boosting and Universal Varicella Vaccination on the Clinical and Economic Burden of Varicella and Herpes Zoster in a Dynamic Population for England and Wales
Source: Vaccines (Basel). 2022 Aug 28;10(9):1416. doi: 10.3390/vaccines10091416 (PMC9501498; doi:10.3390/vaccines10091416)
Supplement: Supplementary file 1 [file vaccines-10-01416-s001.zip › vaccines-1866569-supplementary.pdf]

Supplementary Material

Modelling the impact of exogenous boosting and universal varicella vaccination on the clinical and economic burden of varicella and herpes zoster in a dynamic population for England and Wales

S1 Demographic Model

Several mathematical models of VZV transmission dynamics and HZ reactivation assume a population with a stable age structure.[1-4] However, demographic changes can play an essential role in VZV transmission and reactivation dynamics. Thus, a demographic model with a dynamically changing population age structure is warranted. Consider the Lotka-McKendrick (L-M) model with migration, given below

$$\frac{\partial U}{\partial t}+\frac{\partial U}{\partial a}+\mu(a,t)U = \Upsilon(a,t)U, \qquad 0 \le a < \infty \text{ and } t \ge 0 \qquad (1)$$

$$\Lambda(\mathrm{t}) = U(0,t) = \int_0^\infty f(a,t)U(a,t)da\,, \quad t \ge 0.$$

$$U(a,0) = u_0(a), \quad t \ge 0.$$

where, $U(a,t)$ is the age-specific density of the population, $f(a,t)$ and $\mu(a,t)$ respectively denote age-specific fertility and mortality and $u_0(a)$ is the initial age distribution. The term $\Upsilon(a,t)U(a,t)$ represents a migration process that is supposed to be under control: $\Upsilon(a,t)$ is the characteristic function illustrating intervention restriction on the age-group a. A force of shrinkage, defined as $\varpi(a,t) = \mu(a,t) - \Upsilon(a,t)$ which combines the effect of both mortality and migration, allows us to re-write the L-M model as follows:

$$\frac{\partial U}{\partial t}+\frac{\partial U}{\partial a} = -\varpi(a,t)U, \qquad 0 \le a < \infty \text{ and } t \ge 0$$

$$\Lambda(\mathrm{t}) = U(0,t) = \int_0^\infty f(a,t)U(a,t)da\,, \quad t \ge 0.$$

Note that a solution for $U(a,t)$ can be written in terms of the boundary conditions by considering advancing along its characteristic lines, i.e., the lines in the a-t plane along which $t-a$ is constant[5]:

$$U(a,t) = \begin{cases} U(a-t,0)\exp\left(-\int_0^t \varpi(a-t+\tau,\tau)\,d\tau\right) & t < a \\ \Lambda(t-a)\left(-\int_0^a \varpi(\tau,t-a+\tau)\,d\tau\right) & t \geq a \end{cases} \qquad (2)$$

Varicella and zoster model assumes discrete age bands where the population is divided into N age groups defined by the age intervals $[a_{j-1}, a_j)$, where $a_1 < a_2 < \cdots < a_N = \infty$. The number of individuals $n_j(t)$ at time t in the age (half-open) interval $[a_{j-1}, a_j)$ is the integral of the age distribution function from a_{j-1} to a_j. Then the number of people in the (half-open) age interval $[a_{j-1}, a_j)$ is [1]

$$n_j(t) \equiv \int_{a_{j-1}}^{a_j} U(a,t)da$$

A system of ordinary differential equations can be derived from the partial differential equations for $U(a,t)$, assuming that $\varpi(a,t)$ is piecewise constant in a:

$$\varpi(a,t) = \varpi_j(t) \text{ for } a \in [a_{j-1}, a_j)$$

Differentiate $n_j(t)$ with respect to t, and then use (1):

$$\frac{dn_j}{dt} = \int_{a_{j-1}}^{a_j} \frac{\partial U}{\partial t} da = -\int_{a_{j-1}}^{a_j} \left[\frac{\partial U}{\partial a} + \varpi_j(t)\,U(a,t)\right] da$$

$$= \varpi_j(t)\,n_j(t) - U(a_j,t) + U(a_{j-1},t)$$

The Fundamental Theorem of Calculus is used to evaluate the first term of the integral and the piecewise constancy of $\varpi(a,t)$ to evaluate the first term in the integral. From here, transfer rates between age groups are defined similarly to Hethcote [1], with an added time dependency:

$$d_j(t) \equiv \frac{U(a_j,t)}{n_j(t)}$$

This allows to complete the ordinary differential equations:

$$\frac{dn_j(t)}{dt} = d_{j-1}(t)\,n_{j-1}(t) - \left(\varpi_j(t) + d_j(t)\right)\,n_j(t)$$

A solution (whether analytic or numerical) of the L-M model for $U(a,t)$ is used to compute time-dependent coefficients $d_j(t)$ which represent ageing between compartments in any age-structured compartmental model with discrete age bands, including the MSEIRV model used to model VZV transmission and UVV.

Cohort effects, which represent changes in the population distribution by age over relatively short timescales, are implemented. However, most analytical work on the L-M model focuses on long-run steady-state solutions that feature exponential growth (or decay) in time with a time-constant population distribution by age. Thus, a numerical method [6] to solve the L-M model by stepping along the characteristic lines of equation (2) with a fourth-order explicit Runge-Kutta scheme is used, with a step size of $\delta a = \delta t = h$, as shown in **Figure S1.**

Figure S1. Fourth-order explicit Runge-Kutta scheme

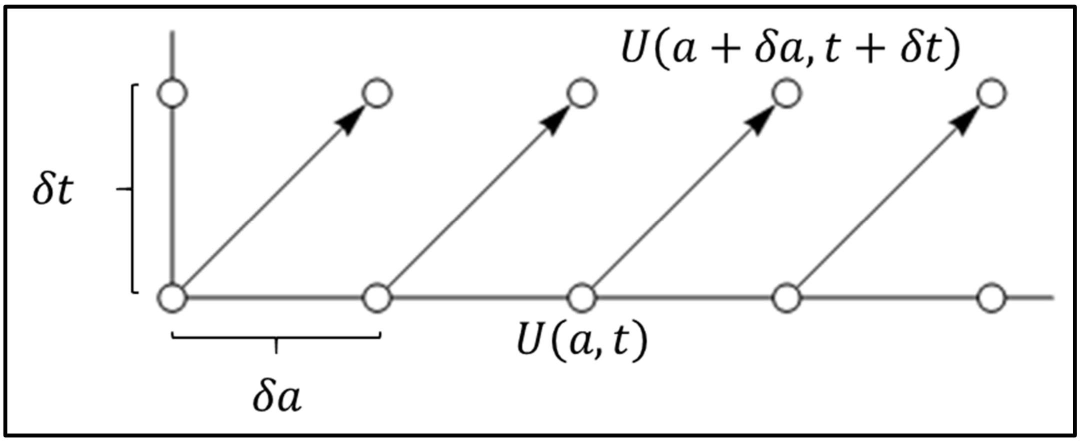

It is then assumed that the age axis is discretized into M steps, for M even, and choose h so that every age group boundary $a_j = l_j h$, where $l_j \in (0, 1, \dots, M)$. Then

$$k_1 = -\varpi(a,t)U(a,t)$$

$$k_2 = -\varpi\left(a + \frac{h}{2}, t + \frac{h}{2}\right)\left[U(a,t) + \frac{h}{2}k_1\right]$$

$$k_3 = -\varpi\left(a + \frac{h}{2}, t + \frac{h}{2}\right)\left[U(a,t) + \frac{h}{2}k_2\right]$$

$$k_4 = -\varpi(a + h, t + h)[U(a,t) + h\, k_3]$$

$$U(a+h,t+h) = U(a,t) + \frac{1}{6}(k_1 + 2k_2 + 2k_3 + k_4) + O(h^5)$$

This allows finding $U\big((l+1)h, t+h\big)$ for $0 \leq l < M$, and Simpson's Rule can be used to evaluate the integral for the total number of births to enforce the boundary condition at $a = 0$:

$$U(0,t+h) = \frac{4h}{3}\sum_{l=0}^{M/2-1} U(a_{2l+1},t)\, f(a_{2l+1},t+h) + \frac{2h}{3}\sum_{l=1}^{M/2-1} U(a_{2l},t) f(a_{2l},t+h)$$

Note that terms for $a = 0$ and $a = Mh$ are omitted because $f(0,t+h) = f(Mh,t+h) = 0$ in a real-world human population.

S1.1 Demographics Inputs and Assumptions

The scheme to solve the L-M model from 1971 through 2122 uses a step size of $h = 0.5$ months, which allows for at least three points to be included in every age group in the model, allowing to use Simpson's rule again to integrate $U(a,t)$, finding $n_j(t)$ and thus $d_j(t)$.

Age-structured demographic inputs were obtained from the UK Office of National Statistics (ONS), using values for England and Wales when available, through to 2018. Mortality rates were available from 1915 through the present [7], data for fertility was available from 1938 onward,[8] and age-structured migration data for England and Wales was available from 1991 onward. [9] When converting these to coefficients in the L-M model with migration, the values were assumed to be piecewise constant in age and were linearly interpolated values between the midpoints of the available time intervals. The model was then run from 1971 on, and the results were validated against age-structured population data for England and Wales, also provided by the ONS. [10] This validation process indicated that in the absence of migration data, an assumption of zero net migration would reproduce the ONS population estimates, so it was assumed that there was no net migration during this period.

To estimate the impact of UVV, the demographic model was also used to project the population size and age structure into the future. The projections were based on ONS assumptions used for their baseline population projections. [11] The assumptions matched were:

1. Between 2018 and 2043, the mortality rate declined 1.2% year-over-year for all persons under age 90, and at 0.6% year over year for persons between 90 and 99 and remained unchanged for persons aged 100 and older.

After 2043, the mortality rate remains constant. These adjustments were applied to the age-structured mortality rates for 2018.

2. Age-structured fertility rates followed those used by the ONS [8] for 2018 through 2043. These were treated identically to the historical fertility rates. After 2043, the rates were assumed to be constant. As there were no separate time series projected fertility rates for England and Wales, we used the projections for the entire UK.

3. Net migration was assumed to change linearly from 2018 to 2025 and remain constant after 2025. Following the ONS assumptions, the migration rates after 2025 were set to the average of the rates over the 25 years from 1993 to 2018. We applied the average separately to each age group from the stratified data.

S2 Model for Varicella and HZ Natural History and Vaccine Effects

Figure S2 shows the diagram of the compartmental model for varicella and HZ natural history and vaccine effects, with the model variables described in Table S1.

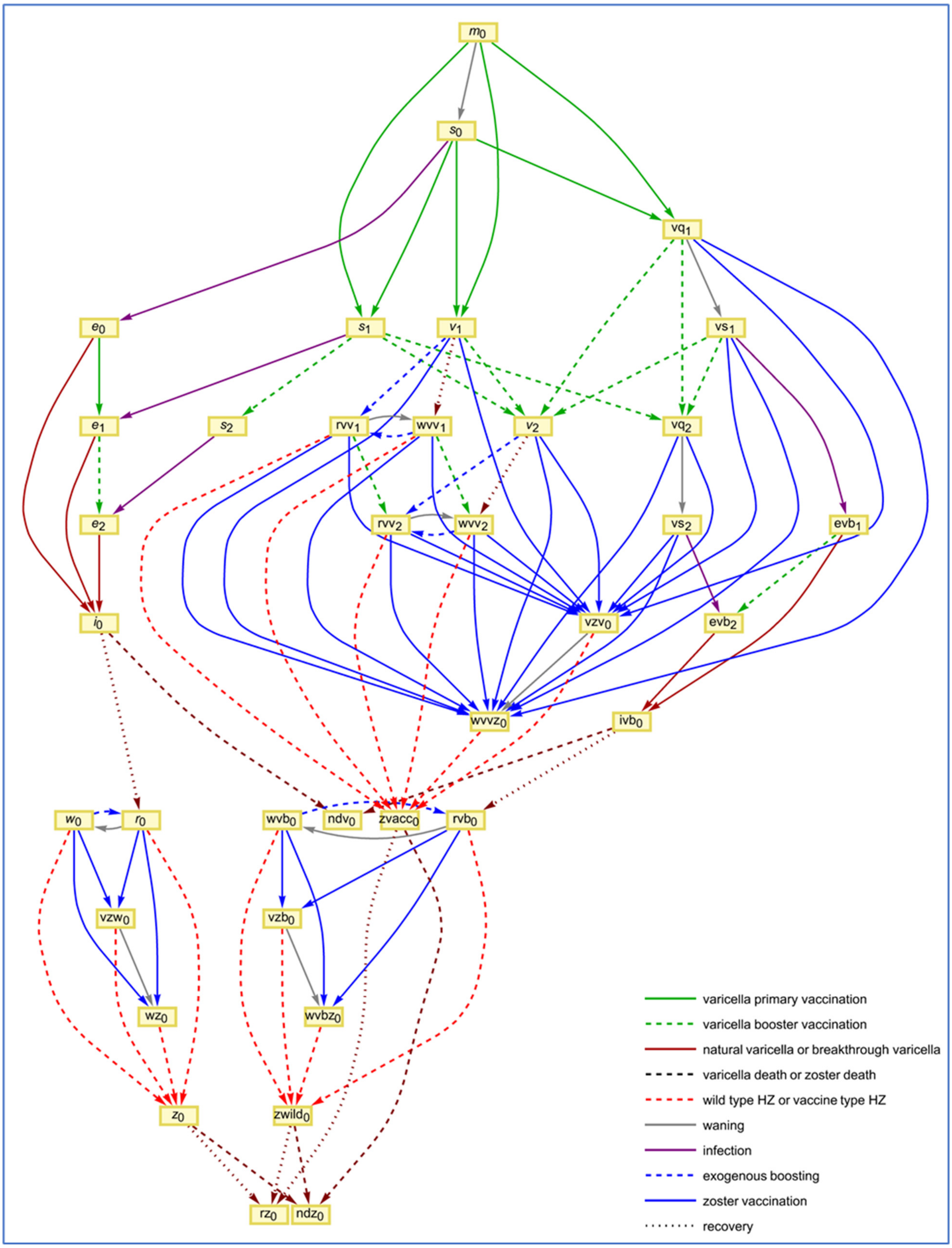

Figure S2. Model diagram for varicella and HZ natural history and vaccine effects.

Table S1. Model variables

Category	Variables	
**Unvaccinated**	m_j	passively immune
	s_j	susceptible to varicella infection
	e_j	latent varicella
	i_j	infectious varicella
	r_j	recovered from varicella with high HZ immunity
	w_j	low HZ immunity due to waning effects
**Varicella vaccinated**	$v_{j,l}$	long-lasting immunity following l −dose vaccination
	$vq_{j,l}$	temporary immunity following l −dose vaccination
	$vs_{j,l}$	susceptible to varicella following l −dose vaccine waning
	$s_{j,l}$	susceptible to varicella following l −dose vaccine failure
	$e_{j,l}$	latent varicella following l −dose vaccine failure
	$rvv_{j,l}$	High HZ immunity following l −dose vaccination
	$wvv_{j,l}$	low HZ immunity following l −dose vaccine waning
**Breakthrough varicella**	$evb_{j,l}$	latent varicella following l −dose vaccine waning
	ivb_j	infectious varicella
	rvb_j	recovered from varicella with high HZ immunity
	wvb_j	low HZ immunity due to waning
**HZ reactivation**	z_j	infectious with wild type HZ
	$zvacc_j$	infectious with wild type HZ post varicella vaccination
	$zwild_j$	infectious with wild type HZ post breakthrough varicella
	rz_j	recovered from HZ with high HZ immunity
**Death**	ndv_j	Death from varicella
	ndz_j	Death from HZ

S2.1 Model Ordinary Differential Equations

$$\frac{dm_j}{dt} = B^m(t)\delta_{1,j} + d_{j-1}(t)(1-\delta_{1,j})\left(1-\theta_j^p(t)-\theta_j^c(t)-\theta_j^s(t)\right)m_{j-1}(t) \ -\left(d_j(t)+\omega^m+\mu_j(t)\right)m_j(t).$$

$$\frac{ds_j}{dt} = B^s(t)\delta_{1,j} + \ \omega^m m_j(t) + d_{j-1}(t)(1-\delta_{1,j})\left(1-\theta_j^p(t)-\theta_j^c(t)-\theta_j^s(t)\right)s_{j-1}(t)$$

$$-\left(d_j(t)+\mu_j(t)+\lambda_j(t)\right)s_j(t).$$

$$\frac{de_j}{dt} = \lambda_j(t)s_j(t) + d_{j-1}(t)(1-\delta_{1,j})\left(1-\theta_j^p(t)-\theta_j^c(t)-\theta_j^s(t)\right)e_{j-1}(t) \ -\left(d_j(t)+\mu_j(t)+\epsilon^n\right)e_j(t).$$

$$\frac{di_j}{dt} = d_{j-1}(t)(1-\delta_{1,j})i_{j-1}(t) + \epsilon^n\left(e_j(t)+ \ e_{j,1}(t)+e_{j,2}(t)\right) - \ \left(d_j(t)+\mu_j(t)+\gamma^n+d_j^v\right)i_j(t).$$

$$\frac{dr_j}{dt} = d_{j-1}(t)(1-\delta_{1,j})r_{j-1}(t) \ +\left({\xi^n}_j+{\zeta^n}_j\lambda_j\right)(t)w_j(t) + \ \gamma^n i_j(t) - \left(d_j(t)+\mu_j(t)+\delta^n+bxz\,\sigma_j\right)r_j(t).$$

$$\frac{dw_j}{dt} = d_{j-1}(t)(1-\delta_{1,j})\left(1-\theta_j^z(t)\right)w_{j-1}(t) + \delta^n r_j(t) - \left(d_j(t)+\mu_j(t)+\sigma_j+{\xi^n}_j+{\zeta^n}_j\lambda_j(t)\right)w_j(t).$$

$$\frac{dv_{j,1}}{dt} = d_{j-1}(t)(1-\delta_{1,j})v_{j-1,1}(t)$$

$$+ d_{j-1}(t)(1-\delta_{1,j})\left[\left(\theta_j^p(t)+\theta_j^c(t)+\theta_j^s(t)\right)PT_1\left(m_{j-1}(t)+s_{j-1}(t)\right)\right.$$

$$\left.-\left(\theta_j^b(t)+\theta_j^d(t)\right)v_{j-1,1}(t)\right] - \left(d_j(t)+\mu_j(t)+\pi_1+k_1\lambda_j(t)\right)v_{j,1}(t).$$

$$\frac{dv_{j,2}}{dt} = d_{j-1}(t)(1-\delta_{1,j})v_{j-1,2}(t)$$

$$+ +d_{j-1}(t)(1-\delta_{1,j})\left(\theta_j^b(t)+\theta_j^d(t)\right)\left[v_{j-1,1}(t)+PT_1 s_{j-1,1}(t)+T_2\left(vq_{j-1,1}(t)+vs_{j-1,1}(t)\right)\right]$$

$$-\left(d_j(t)+\mu_j(t)+\pi_2+k_2\lambda_j(t)\right)v_{j,2}(t).$$

$$\frac{dvq_{j,1}}{dt} = d_{j-1}(t)(1-\delta_{1,j})vq_{j-1,1}(t)$$

$$+ d_{j-1}(t)(1-\delta_{1,j})\left[P(1-T_1)\left(\theta_j^p(t)+\theta_j^c(t)+\theta_j^s(t)\right)\left(m_{j-1}(t)+s_{j-1}(t)\right)\right.$$

$$\left.-\left(\theta_j^b(t)+\theta_j^d(t)+\theta_j^z(t)\right)vq_{j-1,1}(t)\right] - \left(d_j(t)+\mu_j(t)+\sigma^v\right)vq_{j,1}(t).$$

$$\frac{dvq_{j,2}}{dt} = d_{j-1}(t)(1-\delta_{1,j})\left(1-\theta_j^z(t)\right)vq_{j-1,2}(t)$$

$$+ d_{j-1}(t)(1-\delta_{1,j})\left(\theta_j^b(t)+\theta_j^d(t)\right)\left[P(1-T_1)s_{j-1,1}(t)+(1-T_2)\left(vq_{j-1,1}(t)+vs_{j-1,1}(t)\right)\right]$$

$$-\left(d_j(t)+\mu_j(t)+\sigma^v\right)vq_{j,2}(t).$$

$$\frac{dvs_{j,1}}{dt} = \sigma^v vq_{j,1}(t) + d_{j-1}(t)(1-\delta_{1,j})\left(1-\theta_j^b(t)-\theta_j^d(t)-\theta_j^z(t)\right)vs_{j-1,1}(t)$$

$$-\left(d_j(t)+\mu_j(t)+\lambda_j(t)\right)vs_{j,1}(t).$$

$$\frac{dvs_{j,2}}{dt} = \sigma^v vq_{j,2}(t) + d_{j-1}(t)(1-\delta_{1,j})\left(1-\theta_j^z(t)\right)vs_{j-1,2}(t) - \left(d_j(t)+\mu_j(t)+\lambda_j(t)\right)vs_{j,2}(t).$$

$$\frac{dvzv_j}{dt} = d_{j-1}(t)(1-\delta_{1,j})vzv_{j-1}(t)$$

$$- Tzd_{j-1}(t)(1-\delta_{1,j})\theta_j^z(t)\left(vq_{j-1,1}(t)+vq_{j-1,2}(t)+vs_{j-1,1}(t)+vs_{j-1,2}(t)+wvv_{j-1,1}(t)\right.$$

$$\left.+\, wvv_{j-1,2}(t)\right) - \left(\omega\mathrm{z} + d_j(t)+\mu_j(t)+\mathrm{bz}\,\chi\sigma_j(t)\right)vzv_j(t),$$

$$\frac{dvzw_j}{dt} = d_{j-1}(t)(1-\delta_{1,j})\left(vzw_{j-1}(t)+Tz\,\theta_j^z(t)w_{j-1}(t)\right) - \left(\omega^z+d_j(t)+\mu_j(t)+\mathrm{bz}\,\sigma_j(t)\right)vzw_j(t),$$

$$\frac{dvzb_j}{dt} = d_{j-1}(t)(1-\delta_{1,j})\left(vzb_{j-1}(t)+Tz\,\theta_j^z(t)wvb_{j-1}(t)\right) - \left(\omega^z+d_j(t)+\mu_j(t)+\mathrm{bz}\,\chi\,\sigma_j(t)\right)vzb_j(t),$$

$$\frac{ds_{j,1}}{dt} = d_{j-1}(t)(1-\delta_{1,j})s_{j-1,1}(t)$$

$$+ d_{j-1}(t)(1-\delta_{1,j})\left[(1-P)\left(\theta_j^p(t)+\theta_j^c(t)+\theta_j^s(t)\right)\left(m_{j-1}(t)+s_{j-1}(t)\right)\right.$$

$$\left.-\left(\theta_j^b(t)+\theta_j^d(t)\right)s_{j-1,1}(t)\right] - \left(d_j(t)+\mu_j(t)+\lambda_j(t)\right)s_{j,1}(t).$$

$$\frac{ds_{j,2}}{dt} = d_{j-1}(t)(1-\delta_{1,j})s_{j-1,2}(t) + d_{j-1}(t)(1-\delta_{1,j})(1-P)\left(\theta_j^b(t)+\theta_j^d(t)\right)s_{j-1,1}(t)$$

$$-\left(d_j(t)+\mu_j(t)+\lambda_j(t)\right)s_{j,2}(t).$$

$$\frac{de_{j,1}}{dt} = d_{j-1}(t)(1-\delta_{1,j})e_{j-1,1}(t)$$

$$+ d_{j-1}(t)(1-\delta_{1,j})\left[\left(\theta_j^p(t) + \theta_j^c(t) + \theta_j^s(t)\right)e_{j-1}(t) - \left(\theta_j^b(t) + \theta_j^d(t)\right)e_{j-1,1}(t)\right]$$

$$+ \lambda_j(t)s_{j,1}(t) - \left(d_j(t) + \mu_j(t) + \epsilon^n\right)e_{j,1}(t).$$

$$\frac{de_{j,2}}{dt} = d_{j-1}(t)(1-\delta_{1,j})e_{j-1,2}(t) + d_{j-1}(t)(1-\delta_{1,j})\left(\theta_j^b(t) + \theta_j^d(t)\right)e_{j-1,1}(t) + \lambda_j(t)s_{j,2}(t)$$

$$- \left(d_j(t) + \mu_j(t) + \epsilon^n\right)e_{j,2}(t).$$

$$\frac{drvv_{j,1}}{dt} = d_{j-1}(t)(1-\delta_{1,j})\left(1 - \theta_j^b(t) - \theta_j^d(t)\right)rvv_{j-1,1}(t) + \xi^{vv}{}_j wvv_{j,1}(t) + \lambda_j(t)\left[k_1 v_{j,1}(t) + \zeta^{vv}{}_j wvv_{j,1}(t)\right]$$

$$- \left(d_j(t) + \mu_j(t) + bxz\,\chi\sigma_j + \delta^{vv}\right)rvv_{j,1}(t).$$

$$\frac{drvv_{j,2}}{dt} = d_{j-1}(t)(1-\delta_{1,j})\left[\left(\theta_j^b(t) + \theta_j^d(t)\right)rvv_{j-1,1}(t) + rvv_{j-1,2}(t)\right] + \xi^{vv}{}_j wvv_{j,2}(t)$$

$$+ \lambda_j(t)\left[k_2 v_{j,2}(t) + \zeta^{vv}{}_j wvv_{j,2}(t)\right] - \left(d_j(t) + \mu_j(t) + bxz\,\chi\sigma_j + \delta^{vv}\right)rvv_{j,2}(t).$$

$$\frac{dwvv_{j,1}}{dt} = d_{j-1}(t)(1-\delta_{1,j})\left(1 - \theta_j^b(t) - \theta_j^d(t) - \theta_j^z(t)\right)wvv_{j-1,1}(t) + \delta^{vv}rvv_{j,1}(t) + \pi_1 v_{j,1}(t)$$

$$- \left(d_j(t) + \mu_j(t) + \chi\sigma_j + \xi^{vv}{}_j + \zeta^{vv}{}_j\lambda_j(t)\right)wvv_{j,1}(t).$$

$$\frac{dwvv_{j,2}}{dt} = d_{j-1}(t)(1-\delta_{1,j})\left(1 - \theta_j^z(t)\right)wvv_{j-1,2}(t) + d_{j-1}(t)(1-\delta_{1,j})\left(\theta_j^b(t) + \theta_j^d(t)\right)wvv_{j-1,1}(t)$$

$$+ \delta^{vv}rvv_{j,2}(t) + \pi_2 v_{j,2}(t) - \left(d_j(t) + \mu_j(t) + \chi\sigma_j + \xi^{vv}{}_j + \zeta^{vv}{}_j\lambda_j(t)\right)wvv_{j,2}(t).$$

$$\frac{dwz_j}{dt} = \omega^z vzw_j(t) + d_{j-1}(t)(1-\delta_{1,j})\left[(1-Tz)\theta_j^z(t)w_{j-1}(t) + wz_{j-1}(t)\right] - \left(d_j(t) + \mu_j(t) + \sigma_j\right)wz_j(t).$$

$$\frac{devb_{j,1}}{dt} = d_{j-1}(t)(1-\delta_{1,j})\left(1 - \theta_j^b(t) - \theta_j^d(t)\right)evb_{j-1,1}(t) + \lambda_j(t)vs_{j,1}(t) - \left(d_j(t) + \mu_j(t) + \epsilon^{vb}\right)evb_{j,1}(t).$$

$$\frac{devb_{j,2}}{dt} = d_{j-1}(t)(1-\delta_{1,j})evb_{j-1,2}(t) + \lambda_j(t)vs_{j,2}(t) + d_{j-1}(t)(1-\delta_{1,j})\left(\theta_j^b(t) + \theta_j^d(t)\right)evb_{j-1,1}(t)$$

$$- \left(d_j(t) + \mu_j(t) + \epsilon^{vb}\right)evb_{j,2}(t).$$

$$\frac{divb_j}{dt} = d_{j-1}(t)\big(1-\delta_{1,j}\big)ivb_{j-1}(t) + \epsilon^{vb}\left(evb_{j,1}(t) + evb_{j,2}(t)\right) - \big(d_j(t) + \mu_j(t) + \gamma^{vb} + d_j^{vb}\big)ivb_j(t).$$

$$\frac{drvb_j}{dt} = d_{j-1}(t)\big(1-\delta_{1,j}\big)rvb_{j-1}(t) + \left(\xi^{vb}{}_j + \zeta^{vb}{}_j\lambda_j(t)\right)wvb_j(t) + \gamma^{vb}ivb_j(t)$$

$$- \big(d_j(t) + \mu_j(t) + \delta^{vb} + bxz\,\chi\sigma_j\big)rvb_j(t).$$

$$\frac{dwvb_j}{dt} = d_{j-1}(t)\big(1-\delta_{1,j}\big)\left(1-\theta_j^z(t)\right)wvb_{j-1}(t) + \delta^{vb}rvb_j(t)$$

$$- \left(d_j(t) + \mu_j(t) + \chi\sigma_j + \xi^{vb}{}_j + \zeta^{vb}{}_j\lambda_j(t)\right)wvb_j(t).$$

$$\frac{dwvbz_j}{dt} = \omega^z vzb_j(t) + d_{j-1}(t)\big(1-\delta_{1,j}\big)\big[(1-Tz)\theta_j^z(t)wvb_{j-1}(t) + wvbz_{j-1}(t)\big]$$

$$- \big(d_j(t) + \mu_j(t) + \sigma_j\big)wvbz_j(t).$$

$$\frac{dwvvz_j}{dt} = \omega^z vzv_j(t)$$

$$+ d_{j-1}(t)\big(1-\delta_{1,j}\big)\Big[(1-Tz)\theta_j^z(t)\left(vq_{j-1,1}(t) + vq_{j-1,2}(t) + vs_{j-1,1}(t) + vs_{j-1,2}(t)\right.$$

$$\left. + wvv_{j-1,1}(t) + wvv_{j-1,2}(t)\right) + wvvz_{j-1}(t)\Big] - \big(d_j(t) + \mu_j(t) + \chi\sigma_j\big)wvvz_j(t).$$

$$\frac{dz_j}{dt} = \sigma_j\left(bxz\,r_j(t) + bz\,vzw_j(t) + w_j(t) + wz_j(t)\right) + d_{j-1}(t)\big(1-\delta_{1,j}\big)z_{j-1}(t)$$

$$- \big(d_j(t) + \mu_j(t) + \eta^n + d_j^z\big)z_j(t).$$

$$\frac{dzvacc_j}{dt} = d_{j-1}(t)\big(1-\delta_{1,j}\big)zvacc_{j-1}(t)$$

$$+ \chi\sigma_j\left(bxz\,rvv_{j,1}(t) + bxz\,rvv_{j,2}(t) + bz\,vzv_j(t) + wvv_{j,1}(t) + wvv_{j,2}(t) + wvvz_j(t)\right)$$

$$- \big(d_j(t) + \mu_j(t) + \eta^{vv} + d_j^z\big)zvacc_j(t).$$

$$\frac{dzwild_j}{dt} = d_{j-1}(t)\big(1-\delta_{1,j}\big)zwild_{j-1}(t) + \chi\sigma_j\left(bxz\,rvb_j(t) + bz\,vzb_j(t) + wvb_j(t) + wvbz_j(t)\right)$$

$$- \big(d_j(t) + \mu_j(t) + \eta^{vb} + d_j^z\big)zwild_j(t).$$

$$\frac{drz_j}{dt} = d_{j-1}(t)\big(1-\delta_{1,j}\big)rz_{j-1}(t) + \eta^{n}z_j(t) + \eta^{vb}zwild_j(t) + \eta^{vv}zvacc_j(t) - \Big(d_j(t)+\mu_j(t)\Big)rz_j(t).$$

$$\frac{dndv_j}{dt} = d_{j-1}(t)\big(1-\delta_{1,j}\big)ndv_{j-1}(t) + d_j^{v}i_j(t) + d_j^{vb}ivb_j(t) - \Big(d_j(t)+\mu_j(t)\Big)ndv_j(t).$$

$$\frac{dndz_j}{dt} = d_{j-1}(t)\big(1-\delta_{1,j}\big)ndz_{j-1}(t) + d_j^{z}\Big(z_j(t) + zwild_j(t) + zvacc_j(t)\Big) - \Big(d_j(t)+\mu_j(t)\Big)ndz_j(t).$$

$$\lambda_j(t) = \sum_a \beta_{j,a}\big(i_a(t) + \rho^{v}ivb_a(t) + \rho^{z}z_a(t) + \rho^{z}zwild_a(t) + \rho^{z}zvacc_a(t)\big).$$

$$\alpha_j = \begin{cases} rr_1\left(1-\Delta\begin{cases}1 & t<1981\\ 1-\dfrac{t-1981}{1993-1981} & 1981\le t<1993\\ 0 & 1993\le t\end{cases}\right) & a_j<5\\ rr_2 & 5\le a_j<10\\ rr_3 & 10\le a_j<20\\ rr_4 & 20\le a_j\end{cases}$$

$$\sigma_j = \frac{\int_{a_{j-1}}^{a_j} n_j(t)\left(e^{-\phi\,\tau}\omega + \dfrac{\tau^{\eta}\pi}{100000}\right)d\tau}{\int_{a_{j-1}}^{a_j} n_j(t)d\tau}$$

$$B^{m}(t) = \Lambda(\mathrm{t}) - \frac{\Lambda(\mathrm{t})}{\sum_j f_j(t)n_j}\sum_j\left(f_j(t)s_j(t) + \sum_l f_j(t)s_{j,l}(t)\right).$$

$$B^{s}(t) = \frac{\Lambda(\mathrm{t})}{\sum_j f_j(t)n_j}\sum_j\left(f_j(t)s_j(t) + \sum_l f_j(t)s_{j,l}(t)\right).$$

$$\Lambda(\mathrm{t}) = U(0,t) = \int_0^{\infty} f(a,t)U(a,t)da\,,\quad t\ge 0.$$

$$d_j(t) \equiv \frac{U\big(a_j,t\big)}{\int_{a_{j-1}}^{a_j} U(a,t)da}$$

$$U(a,t) = \begin{cases} U(a-t,0)\exp\left(-\int_0^t \varpi(a-t+\tau,\tau)\,d\tau\right) & t < a \\ \Lambda(t-a)\left(-\int_0^a \varpi(\tau, t-a+\tau)\,d\tau\right) & t \geq a \end{cases}$$

S2.2 Definition of vaccination coverage

No vaccination strategy:

$$\theta_j^p(t) = \theta_j^c(t) = \theta_j^b(t) = \theta_j^d(t) = 0$$

Single dose strategy:

$$\begin{aligned} \theta_j^p(t) &= \delta_{j,13}\phi^p && t \geq 0. \\ \theta_j^c(t) &= 0 && t \geq 0. \\ \theta_j^b(t) &= 0 && t \geq 0. \\ \theta_j^d(t) &= 0 && t \geq 0. \end{aligned}$$

2-dose short interval without catch up:

$$\begin{aligned} \theta_j^p(t) &= \delta_{j,7}\phi^p && t \geq 0. \\ \theta_j^c(t) &= 0 && t \geq 0. \\ \theta_j^b(t) &= \begin{cases} 0 \\ \delta_{j,13}\phi^{bs} \end{cases} && \begin{matrix} t < 6 \text{ months}, \\ t \geq 6 \text{ months}. \end{matrix} \\ \theta_j^d(t) &= 0 && t \geq 0. \end{aligned}$$

2-dose short interval with catch up:

$$\begin{aligned} \theta_j^p(t) &= \delta_{j,7}\phi^p && t \geq 0. \\ \theta_j^c(t) &= \delta_{j,33}\phi^c && 0 \leq t \leq 1. \\ \theta_j^b(t) &= \begin{cases} 0 \\ \delta_{j,13}\phi^{bs} \end{cases} && \begin{matrix} t < 6 \text{ months}, \\ t \geq 6 \text{ months}. \end{matrix} \\ \theta_j^d(t) &= \delta_{j,34}\phi^d && 1 \leq t \leq 2. \end{aligned}$$

2-dose medium interval with catch up:

$$\begin{aligned} \theta_j^p(t) &= \delta_{j,7}\phi^p && t \geq 0. \\ \theta_j^c(t) &= \delta_{j,33}\phi^c && 0 \leq t \leq 1. \\ \theta_j^b(t) &= \begin{cases} 0 \\ \delta_{j,21}\phi^{bl} \end{cases} && \begin{matrix} t < 22 \text{ months}, \\ t \geq 22 \text{ months}. \end{matrix} \\ \theta_j^d(t) &= \delta_{j,34}\phi^d && 1 \leq t \leq 2. \end{aligned}$$

The values of ϕ refer to the different coverage rates.

S3 Calibration

In the first phase of the calibration, the model is fitted to historical population sizes and age distribution so that the model reproduces the dynamically changing population age structure (Figure S3).

Figure S3. Observed and calibrated population age structure for selected years

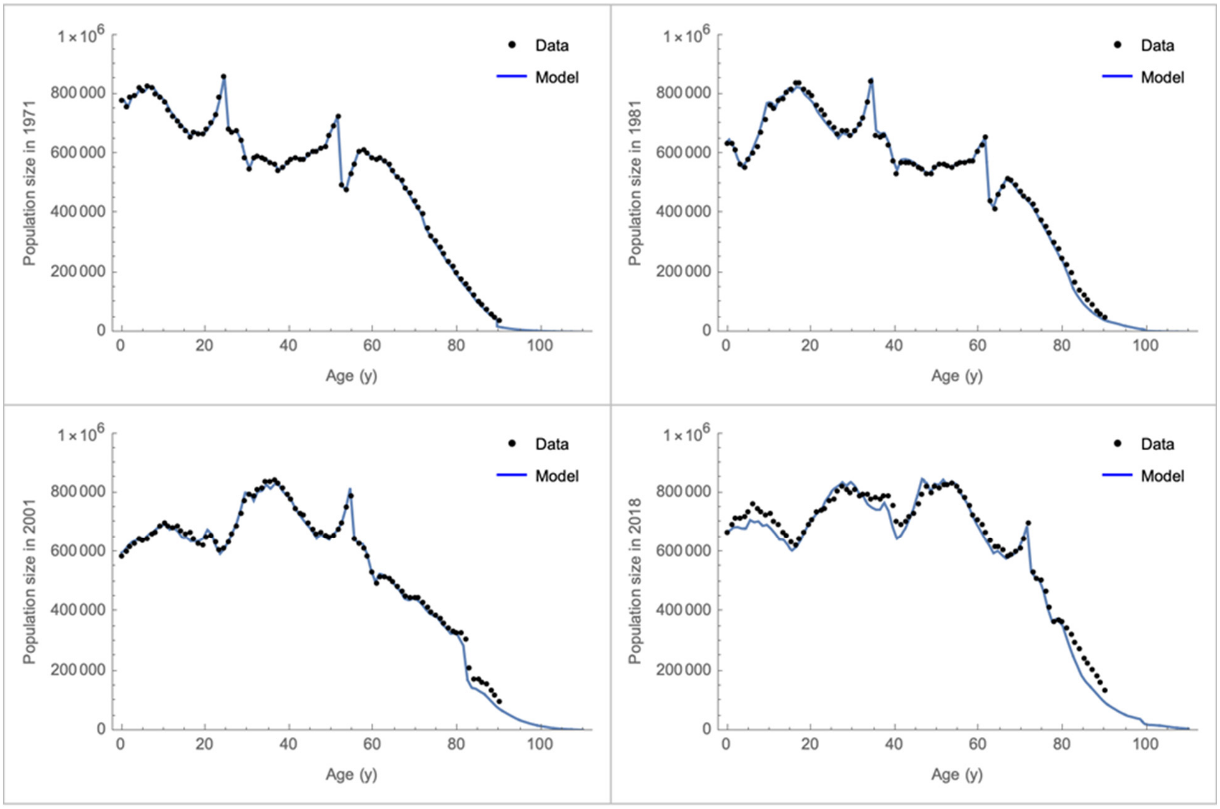

The model was calibrated to age-stratified VZV seroprevalence and HZ incidence to represent the pre-vaccination status quo (Figure S4 and Figure S5). VZV seroprevalence was a preferred target for calibration as most varicella cases do not seek health care and therefore, reliable data on varicella incidence is unavailable. [2] For example, in the case of the UK, it is estimated that only 20% of varicella cases among 5- to 14-year-olds require GP visits. [12] Age-stratified seroprevalence provides information about the ages at which people contract varicella [2, 13, 14] and is often used as a calibration target in VZV models. [15] For HZ, however, the incidence is used because it always results in patients seeking care,[12] meaning multiple reliable studies are available for UK.[16-18]

The calibration process involved estimating certain parameters governing the transmission of VZV infection and the natural history of HZ outbreaks. This was done in three steps. First, VZV transmission was calibrated to data on seroprevalence covering periods from 1972 [19] through 2017 [13] using predefined values for the reactivation rate of HZ from the literature. [2] We excluded seroprevalence data from 1968 from Kudesia et al. [19] as only this study had seroprevalence data for that year. Second, HZ reactivation was calibrated to incidence data covering 1986 to 2006 using the values for VZV transmission obtained from the first step. Because the data sources for HZ incidence covered extended periods without being stratified by year, this fit was performed around assumed demographics for 1995. Third, we re-calibrated VZV transmission using the values for HZ reactivation from second step. This process being required given the assumption that persons experiencing HZ outbreaks can transmit varicella infections, though, the probability that they do so is small (relative risk of 7%).

We estimated age-specific risk of transmission rr_1, rr_2, rr_3 , and rr_4 (for ages 5-9, 10-19, and ≥20 years old) for each contact with an infectious person. [12] Additionally, the risk for children under 5 of contracting varicella upon contact with infected individuals was scaled by a time-dependent factor (Δ), with the risk of transmission prior to 1981 being lower relative to the risk after 1993 and increasing linearly between 1981 and 1993. Indeed, a shift in the age at which children contract varicella [18] was observed in England and Wales, with many more children becoming infected before 5 years of age5 starting in the early 1990s, while it had remained relatively stable until that point. Similar effects were observed during the 1980s and 1990s in other developed nations, [19, 20] and they have generally been attributed to increased utilization of day care services. [21] For HZ reactivation, a standard time-independent functional form was used that relates reactivation rate to age. [2]

Likelihood functions for VZV seroprevalence were based on the binomial distribution with the number of trials equal to the reported number of samples for each age group and year. HZ incidence data was based on the results of extensive registry studies and cases over the person-years at risk were approximated with normal distributions.

Figure S4. Observed and fitted VZV seroprevalence data for 1978, 1992, 2004 and 2007

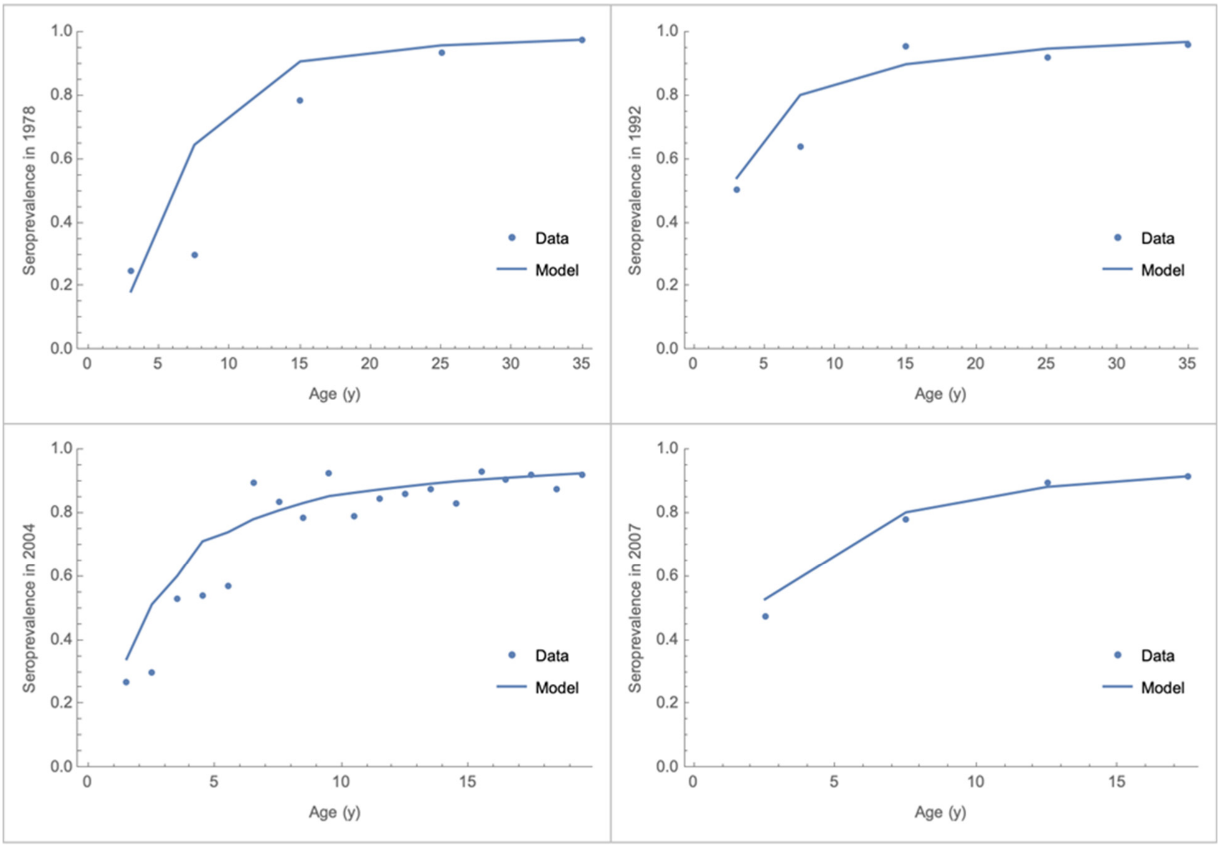

Figure S5. Observed and fitted HZ incidence data

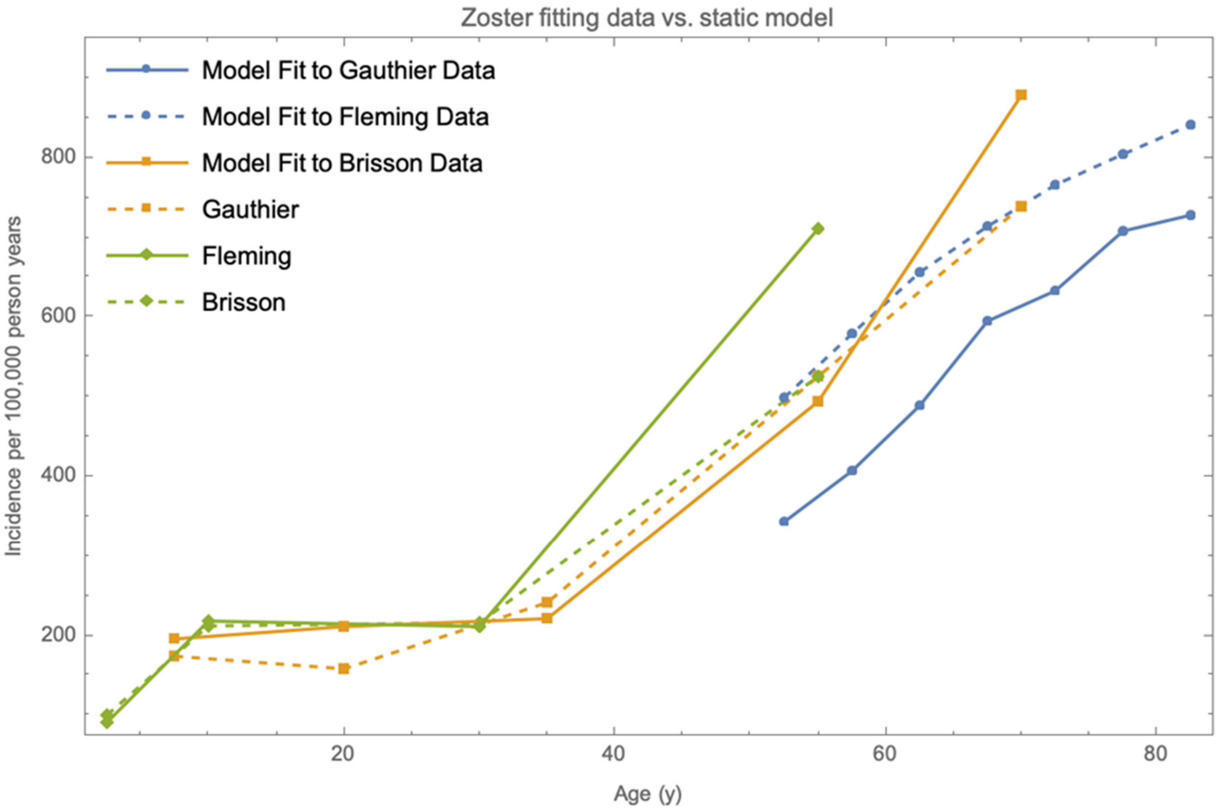

S4 Vaccine Properties

Table S2. Vaccine properties

Parameters		Dose	MSD vaccines	GSK vaccines	Source
$1 - P$	vaccine failure rate	1 & 2	4%	5%	[3, 22]
		1	90.3%	61.7%	[23]
T_l	dose take rate	2	69.0%	83.4%	[23]
		1 & 2	97.0%	93.8%	[23]
$1/_{\sigma^v}$	average duration of protection	1 & 2	1.2 years	0.9 years	[23]
$1/_{\pi_l}$	Average waning period of high HZ immunity following vaccination	1	81.3 years	81.3 years	[24]*
		2	81.3 years	81.3 years	

* Details given in 6.1

S5 Vaccination Strategies

Table S3. Summary of vaccination strategies

Strategy		Formulation		Age at vaccination, months		Vaccination coverage, %		2-dose catch-up at 13 and 14 years old	
		1st dose	2nd dose	1st dose	2nd dose	1st dose	2nd dose	Formulation	Coverage, %
1-dose only	A	MMRV-MSD		18	-	91	-	-	-
	B	MMRV-GSK		18	-	91	-	-	-
2-dose, short interval	C	V-MSD	V-MSD	12	18	91	91	V-MSD	87
	D	V-GSK	V-GSK	12	18	91	91	V-GSK	87
	E	V-MSD	MMRV-MSD	12	18	91	91	-	-
	F	V-GSK	MMRV-GSK	12	18	91	91	-	-
2-dose, medium interval	G	V-MSD	MMRV-MSD	12	40	91	88	V-MSD	87
	H	V-GSK	MMRV-GSK	12	40	91	88	V-GSK	87
	I	V-MSD	V-MSD	12	40	91	88	V-MSD	87
	J	V-GSK	V-GSK	12	40	91	88	V-GSK	87

V-MSD: Varivax, V-GSK: Varilrix, MMRV-MSD: ProQuad, MMRV-GSK: Priorix-Tetra

Note: Coverage rates for the 1st dose are applied to vaccine naïve children who continue to have maternal protection or who remain susceptible for VZV infection; coverage rates for the 2nd dose are applied to children who have not yet been infected with VZV regardless of whether they had received a 1st dose.

S6 Derivation of Model Parameter Inputs

S6.1 Estimation of Boosting Proportion and Duration of Boosting

Forbes estimated the relative incidence of HZ in the 20 years after exposure to a child with varicella in the household compared with baseline time (Table S4) [24].

Table S4. Incidence ratio of HZ following household exposure to a child with varicella

Post-Exposure Risk Period (years)	Mid-Point (years)	Incidence Ratio
0–<2	1	0.67
2–<5	2.5	0.69
5–<10	7.5	0.69
10–20	15	0.73

Using data from Forbes et al. [24], we designed a simple model which attempts to estimate two parameters of the natural history of HZ vis-à-vis the proportion of individuals been boosted (ζ) and the duration of boosting (1/δn). Following recovery from varicella infection, individuals acquire a temporary immunity to HZ (r). Once immunity to HZ has waned, individuals become susceptible to HZ (w) at a rate δn. Individuals can have a reactivation episode at rate σ. The simple model is given below as

$$r'(t) = -\delta\mathrm{n}\, r(t),$$

$$w'(t) = \delta\mathrm{n}\, r(t) - \sigma\, w(t),$$

$$r(0) = \zeta, \qquad w(0) = 1 - \zeta$$

The simple model assumes mortality and ageing have little influence on boosting and its duration. The goal is to use the simple model to compute the analytical incidence ratio and fit the Forbes data [24]. The solution for the number of individuals susceptible to HZ when boosting is

$$w(t,\zeta) = \frac{(\delta\mathrm{n} - (1-\zeta)\sigma)e^{-\sigma t} - \delta\mathrm{n}\zeta e^{-\delta\mathrm{n} t}}{\delta\mathrm{n} - \sigma}.$$

The number of individuals susceptible to HZ without boosting is

$$w(t,0) = e^{-\sigma t}.$$

The model incidence ratio is obtained by dividing the HZ incidence when boosting is active by HZ incidence without boosting. The model incidence ratio is given by

$$\frac{\delta\text{n} - (1-\zeta)\sigma - e^{t(-\delta\text{n}+\sigma)}\delta\text{n}\zeta}{\delta\text{n} - \sigma}.$$

Using reactivation rate $\sigma = e^{-a\phi}\omega + \frac{a^{\eta}\pi}{100000}$ and the associated values from Brisson (2002), i.e., $\pi = 1.06, \eta = 1.91, \omega = 0.11, \phi = 0.17$ and $a = 38.3$, the estimated average value for σ over the next 20 years is $\sigma = 0.0177$. Next, using the least-square method, the values $\delta\text{n} = 0.012299$ and $\zeta = 0.334499$ are obtained by minimizing the squared error between the model incidence ratio and the incidence ratio provided in the Forbes paper (also provided in Table S4). Hence duration of boosting is 1/0.012299= 81.3 years. The least-square fit is depicted below in Figure S6.

Figure S6. Least square fit of the incidence ratio

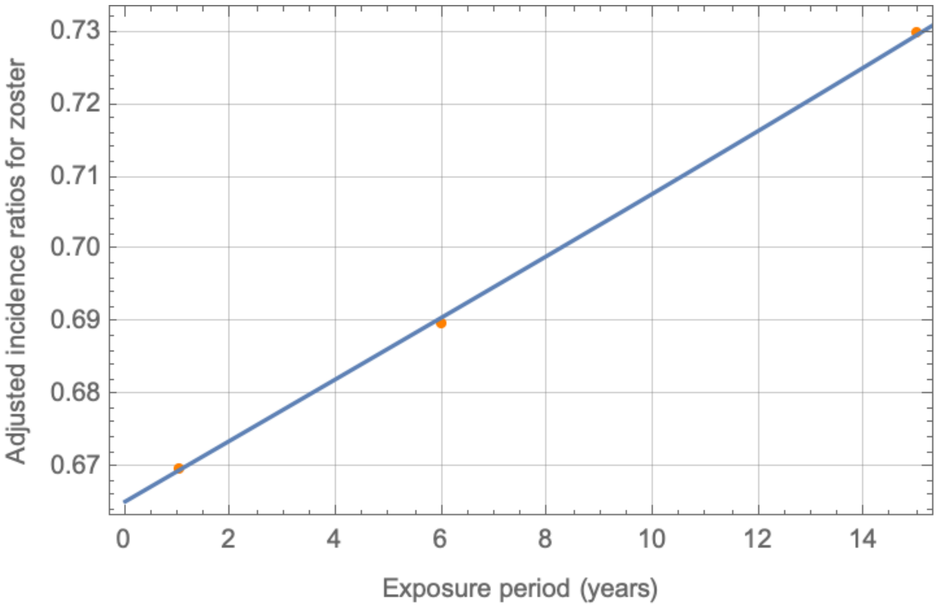

S6.2 GP Visits and Hospitalization for Varicella and HZ

To estimate the proportion of Varicella GP visits, the Varicella consultation rate by age for each ethnic group in England and Wales, as summarized by Walker et al. [25], is used to fit the model projected consultation rate by age. The rate of consultation for varicella (reproduced below in Figure S7) was digitized and reported in Table S5.

Figure S7. Varicella consultation rate*

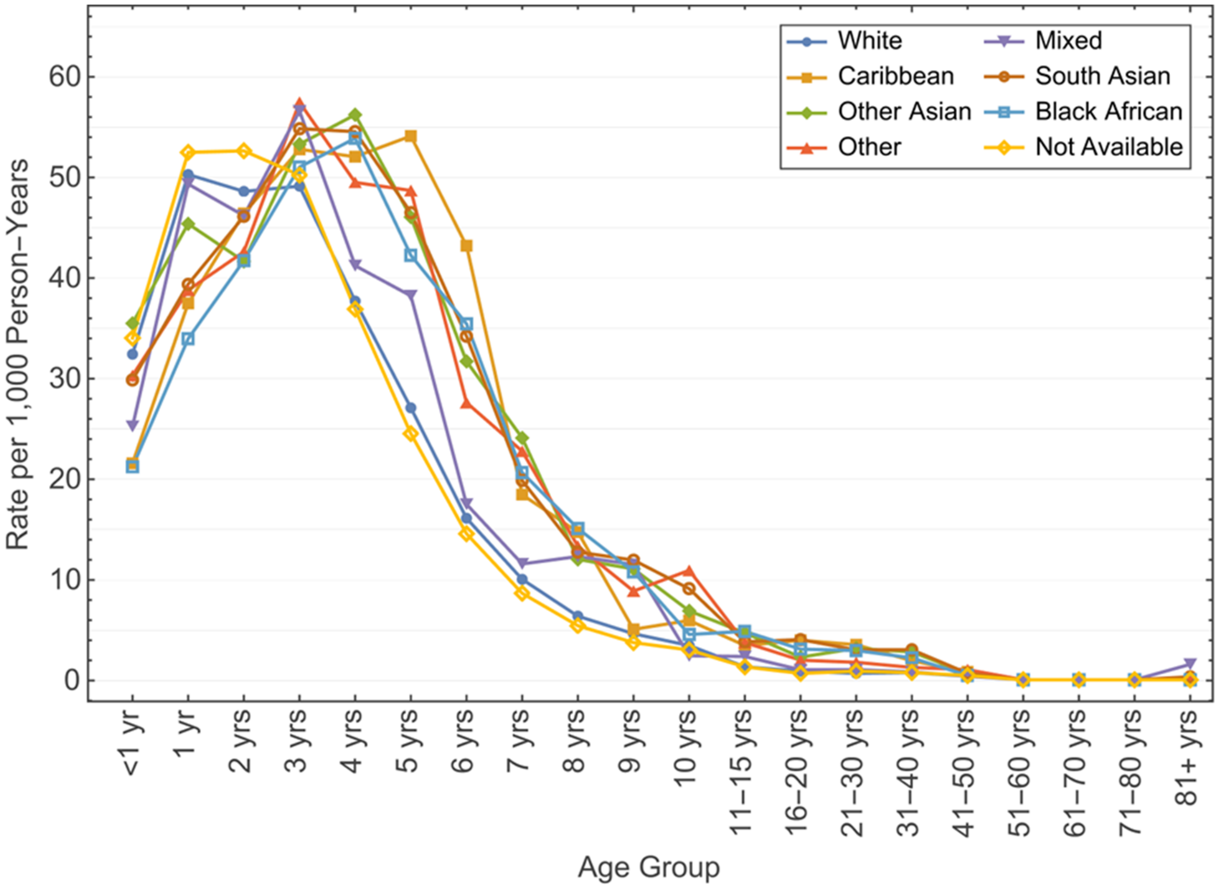

*Source: Walker et al. [25]

Table S5. Digitized varicella consultation rate

Proportions	0.859	0.019	0.014	0.0075	0.053	0.022	0.018	0.0075	1
Year of Age	White	Caribb ean	Other Asian	Other	Mixed	South Asian	Black African	Not Availa ble	All
<1	32.43	21.59	35.50	30.31	25.25	29.87	21.23	34.04	31.62
1	50.29	37.48	45.39	38.80	49.34	39.39	33.97	52.49	49.33
2	48.61	46.42	41.66	42.61	46.20	46.12	41.73	52.64	48.15
3	49.13	52.79	53.30	57.47	56.60	54.84	51.03	50.23	49.88
4	37.71	52.06	56.23	49.50	41.23	54.55	53.89	36.91	39.18
5	27.10	54.11	46.06	48.70	38.23	46.50	42.26	24.54	29.31
6	16.13	43.21	31.72	27.62	17.52	34.21	35.45	14.59	17.76
7	10.06	18.47	24.11	22.79	11.59	19.87	20.67	8.67	10.99
8	6.40	14.75	12.04	13.35	12.33	12.77	15.11	5.45	7.29
9	4.65	5.09	11.09	8.89	11.53	11.97	10.80	3.77	5.41
10	3.48	5.97	6.92	10.95	2.46	9.12	4.58	3.04	3.72
11-15	1.36	3.48	4.73	3.78	2.39	3.85	4.88	1.36	1.64
16-20	0.93	4.00	2.32	2.02	1.07	4.07	3.12	0.71	1.13
21-30	0.71	3.56	3.20	1.81	1.08	3.05	2.98	0.93	0.92
31-40	0.79	1.96	2.84	1.30	0.86	3.06	2.25	0.79	0.92
41-50	0.43	0.86	0.72	1.08	0.43	0.72	0.50	0.50	0.45
51-60	0.07	0.07	0.07	0.07	0.07	0.07	0.07	0.07	0.07
61-70	0.07	0.07	0.07	0.07	0.07	0.07	0.07	0.07	0.07
71-80	0.07	0.07	0.07	0.07	0.07	0.07	0.07	0.07	0.07
≥81	0.07	0.07	0.07	0.07	1.61	0.37	0.07	0.07	0.16

and using the proportions of each ethnic group as weights, a weighted average of the ethnic consultation rates is used to estimate the combined consultation rate by age for all of England and Wales (located in the last column of Table S5). Since Varicella consultation rate is the Proportion of GP visits per case (Proportion of GP visits multiplied by the number of GP visits per case) multiplied by the Varicella incidence rate, the consultation rate is aligned to an exponential function of age so that for each age group defined in the model, the Proportions of GP visits ($V_{GPV}(a)$) can be estimated using the following:

$$V_{GPV}(a) = \frac{V_c(a)}{\text{nGPv(a) x } V_r\text{(a)}}$$

where the number of GP visits per case (nGPv(a)) is provided in Table S6 and model Varicella incidence rate (V_r(a)) is an output of the model with the Varicella consultation rate fit function $V_c(a)$ defined as

$$V_c(a) = 0.09193 + e^{4.35342a^{0.40067} - 0.93362a},$$

and a is the age group index interpolated from index 1 through 20. The fit is depicted below in Figure S8, while the estimated proportion of GP visits is in Table S7.

Table S6. Varicella, HZ and PHN health care resource utilization related parameters

Parameters	Age group, years											
	< 1	[1,5)	[5,15)	[15,25)	[25,45)	[45,60)	[60,65)	[65,70)	[70,75)	[75,80)	[80,85)	≥ 85
Varicella-related care												
percent of cases seeking care	See Table S7											
number of GP visits per case seeking care	1.40	1.41	1.50	1.69	1.66	1.81	1.81	1.69	1.69	1.73	1.73	1.73
percent of patients hospitalized	See Table S7											
days of stay per admission	1.50	1.80	2.50	2.70	4.40	7.70	7.70	9.30	9.30	14.30	14.30	14.30
HZ-related care												
number GP visit per case	1.07	1.07	1.18	1.36	1.36	1.43	1.43	1.68	1.68	1.68	1.68	1.68
percent of patients hospitalized	See Table S7											
days of stay per admission	1.50	1.60	1.10	3.90	4.70	5.10	9.30	8.20	10.80	13.90	17.10	22.30
percent of patients with PHN	0.00	0.00	6.28	6.28	6.28	15.75	15.75	18.80	18.80	18.80	18.80	18.80

Figure S8. Incidence of varicella consultation rate fit

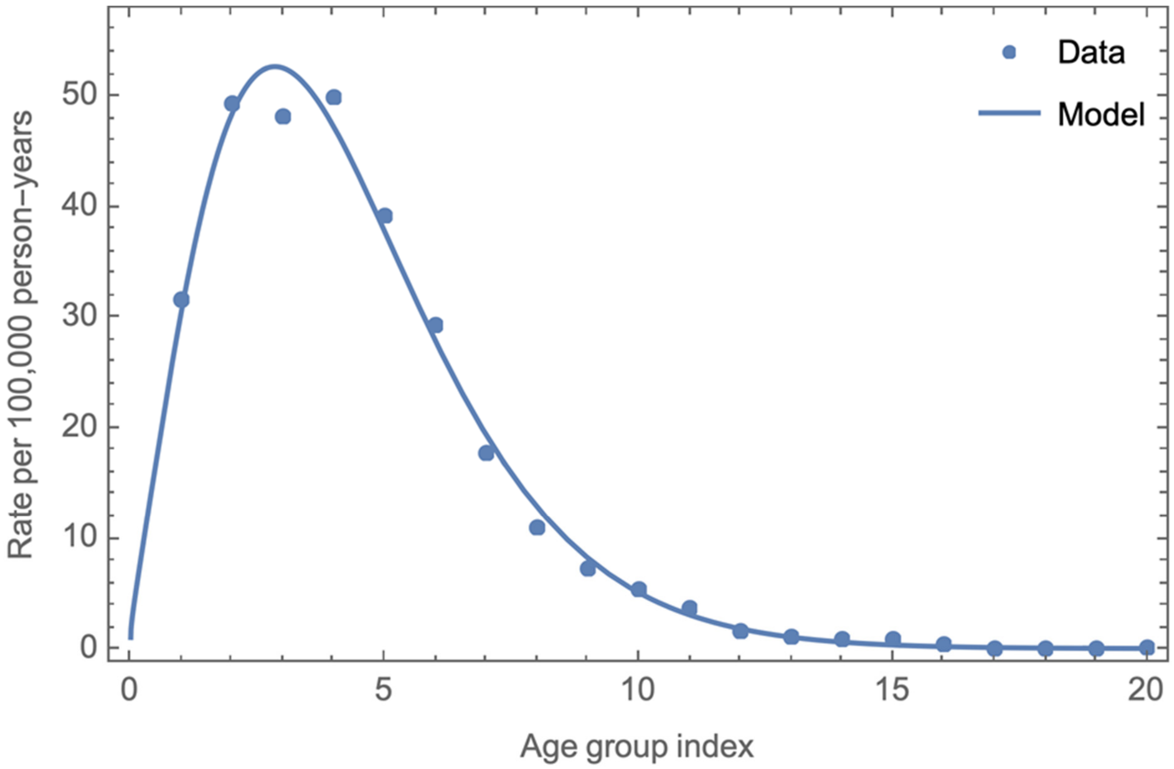

Table S7. Percentage of VZV-related events seeking health care resources

Age group	Age category	% of varicella cases seeking outpatient care	% of varicella cases admitted to hospital	% of HZ cases admitted to hospital
1	[0, 1) months	61.2%	2.0%	100.0%
2	[1, 3) months	25.8%	0.8%	100.0%
3	[3, 6) months	18.0%	0.5%	48.3%
4	[6, 9) months	16.1%	0.4%	20.7%
5	[9, 11) months	16.1%	0.4%	14.2%
6	[11, 12) months	16.6%	0.4%	12.1%
7	[12, 13) months	16.9%	0.3%	9.8%
8	[13, 14) months	17.0%	0.3%	8.6%
9	[14, 15) months	17.2%	0.3%	7.5%
10	[15, 16) months	17.4%	0.3%	6.7%
11	[16, 17) months	17.6%	0.3%	6.0%
12	[17, 18) months	17.9%	0.3%	5.4%
13	[18, 19) months	18.2%	0.3%	4.9%
14	[19, 20) months	18.5%	0.3%	4.5%
15	[20, 21) months	18.8%	0.3%	4.1%
16	[21, 22) months	19.1%	0.3%	3.8%
17	[22, 23) months	19.4%	0.3%	3.5%
18	[23, 24) months	19.7%	0.2%	3.3%
19	[24, 36) months	24.8%	0.3%	1.6%

20	[36, 40) months	24.3%	0.3%	1.5%
21	[40,48) months	26.2%	0.3%	1.2%
22	[4, 5) years	30.1%	0.3%	1.0%
23	[5, 5.5) years	42.8%	0.3%	1.4%
24	[5.5, 6) years	41.3%	0.3%	1.3%
25	[6, 7) years	39.4%	0.2%	1.2%
26	[7, 8) years	30.0%	0.2%	1.2%
27	[8, 9) years	22.1%	0.1%	1.2%
28	[9, 10) years	15.8%	0.1%	1.2%
29	[10, 11) years	18.0%	0.1%	1.3%
30	[11, 12) years	12.1%	0.1%	1.3%
31	[12, 13) years	13.1%	0.1%	1.4%
32	[13, 14) years	14.1%	0.1%	1.5%
33	[14, 15) years	15.3%	0.1%	1.6%
34	[15, 16) years	19.2%	0.2%	0.5%
35	[16, 17) years	12.4%	0.2%	0.5%
36	[17, 18) years	13.1%	0.2%	0.6%
37	[18, 19) years	13.9%	0.2%	0.6%
38	[19, 20) years	14.8%	0.2%	0.6%
39	[20, 25) years	15.2%	0.4%	0.6%
40	[25, 30) years	11.7%	0.2%	0.5%
41	[30, 35) years	12.7%	0.3%	0.5%
42	[35, 40) years	14.4%	0.3%	0.4%
43	[40, 45) years	17.9%	0.6%	0.4%
44	[45, 50) years	31.5%	0.7%	0.3%
45	[50, 55) years	31.1%	1.0%	0.3%
46	[55, 60) years	33.6%	1.1%	0.3%
47	[60, 65) years	69.8%	3.0%	0.2%
48	[65, 70) years	60.9%	2.0%	0.3%
49	[70, 75) years	60.3%	2.4%	0.2%
50	[75, 80) years	100.0%	4.2%	0.2%
51	[80, 85) years	100.0%	4.4%	0.3%
52	[85, 90) years	100.0%	4.6%	0.3%
53	90+ years	100.0%	4.7%	0.5%

A similar approach used to estimate GP proportions is used to estimate the proportion of hospitalization visits for varicella and HZ. In addition, the incidence of hospitalization for varicella and HZ (by HES, according to Hobbelen [26]), also reproduced below in Figure S9, was digitized (see Table S8).

Figure S9. Incidence of Varicella and HZ Hospitalization by age [26]

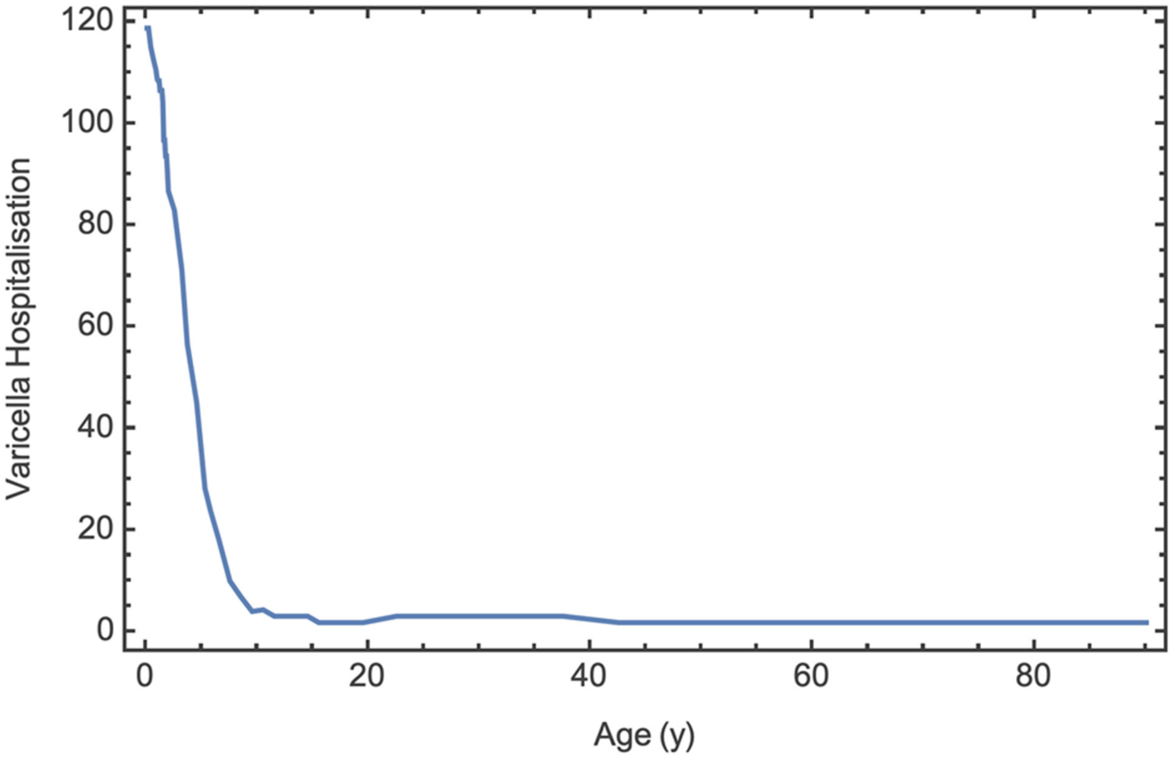

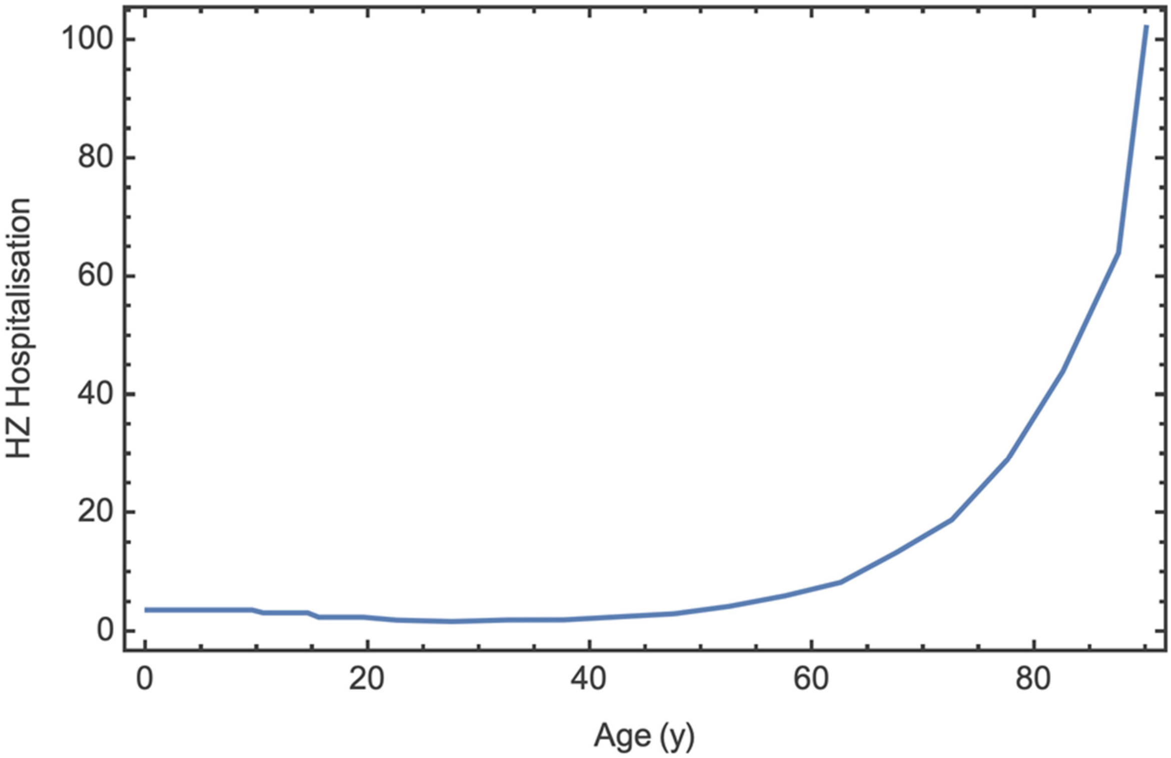

Table S8. Digitized Incidence of Hospitalization for Varicella and HZ

Age group	Age Category	Varicella Hospitalization	HZ Hospitalization
1	[0, 1) months	118.868	3.768
2	[1, 3) months	118.868	3.768
3	[3, 6) months	115.094	3.768
4	[6, 9) months	112.579	3.768
5	[9, 11) months	110.692	3.768
6	[11, 12) months	108.805	3.768
7	[12, 13) months	108.491	3.768
8	[13, 14) months	108.491	3.768
9	[14, 15) months	106.604	3.768
10	[15, 16) months	106.604	3.768
11	[16, 17) months	106.604	3.768
12	[17, 18) months	104.403	3.768
13	[18, 19) months	96.855	3.768
14	[19, 20) months	96.855	3.768
15	[20, 21) months	93.711	3.768
16	[21, 22) months	93.711	3.768
17	[22, 23) months	90.566	3.768
18	[23, 24) months	86.792	3.768
19	[24, 36) months	83.019	3.768
20	[36, 40) months	71.384	3.768
21	[40,48) months	56.604	3.768
22	[4, 5) years	45.283	3.768
23	[5, 5.5) years	28.302	3.768
24	[5.5, 6) years	23.899	3.768
25	[6, 7) years	18.239	3.768
26	[7, 8) years	10.063	3.768
27	[8, 9) years	6.918	3.768
28	[9, 10) years	4.088	3.768
29	[10, 11) years	4.403	3.289
30	[11, 12) years	3.145	3.289
31	[12, 13) years	3.145	3.289
32	[13, 14) years	3.145	3.289
33	[14, 15) years	3.145	3.289
34	[15, 16) years	1.887	2.553
35	[16, 17) years	1.887	2.553
36	[17, 18) years	1.887	2.553
37	[18, 19) years	1.887	2.553
38	[19, 20) years	1.887	2.553
39	[20, 25) years	3.145	2.066
40	[25, 30) years	3.145	1.829
41	[30, 35) years	3.145	2.094
42	[35, 40) years	3.145	2.107
43	[40, 45) years	1.887	2.621
44	[45, 50) years	1.887	3.136
45	[50, 55) years	1.887	4.398
46	[55, 60) years	1.887	6.163
47	[60, 65) years	1.887	8.426
48	[65, 70) years	1.887	13.442
49	[70, 75) years	1.887	18.954
50	[75, 80) years	1.887	29.094

51	[80, 85) years	1.887	44.108
52	[85, 90) years	1.887	64.122
53	90+ years	1.887	102.261

The fitted functions for the incidence of hospitalization for varicella ($V_h(a)$) and HZ ($Z_h(a)$), is given by

$$V_h(a) = 2.60173 + e^{4.67168-6.415\times10^{-9}a^{6.07558}}$$

and

$$Z_h(a) = 1.9072 + e^{1.22842\times10^{-10}a^{6.13337}}$$

so that the proportions of hospitalization visits for varicella ($V_{HospV}(a)$) and HZ ($Z_{HospV}(a)$), is given by

$$V_{HospV}(a) = \frac{V_h(a)}{\text{lsHv(a) x } V_i(\text{a})},$$

$$Z_{HospV}(a) = \frac{Z_h(a)}{\text{lsHz(a) x } Z_i(\text{a})},$$

where lsHv(a) and lsHz(a) represents the length of stay at the hospital for varicella and HZ, respectively, and can be found in Table S6, V_i(a), represents the model's projected incidence of varicella and Z_i(a), represents the model's projected incidence of HZ. The alignment between the functions and the data is depicted in Figure S10 and Figure S11. The estimated proportions of hospitalization for varicella and HZ can be found in Table S7.

Figure S10. Incidence of varicella hospitalization fit

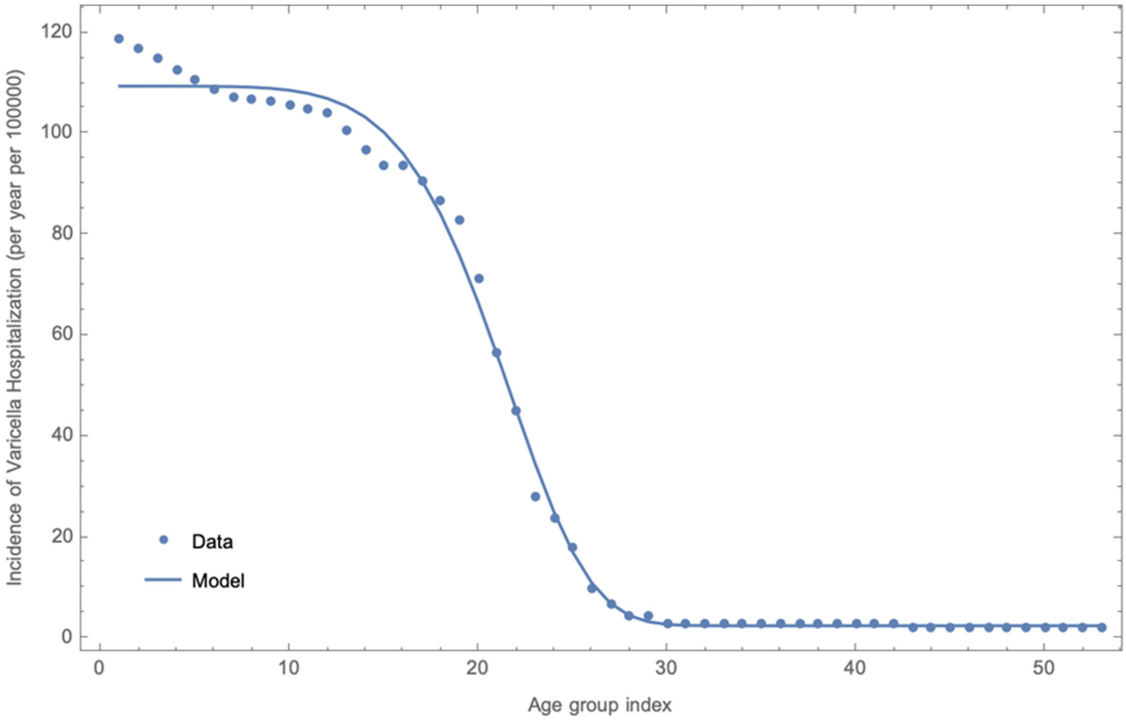

Figure S11. Incidence of HZ hospitalization fit

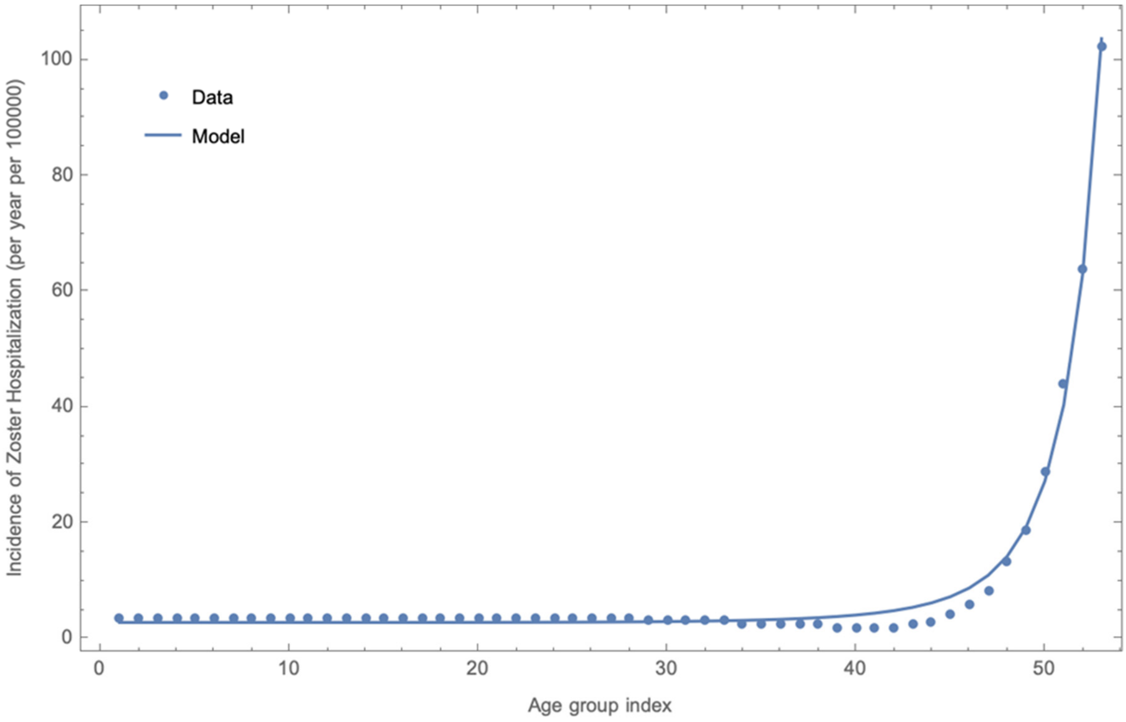

S6.3 Case Fatality Rate

Varicella and HZ death are fitted to mortality data summarized in Brisson and Edmunds (2003) through calibration [28]. The new case fatality rates and fits are displayed below in Table S9, and Figure S12 (varicella) and Figure S13 (HZ).

Table S9. Case fatality rates (per 100,000 patient years)

	Age Group					Source
	0-5	5-15	15-45	45-65	65+	
Natural varicella	0.63	0.67	19.83	144.52	1545.49	Calibrated
Breakthrough varicella	0.2	0.2	1.8	14.6	137.8	[2]
HZ	0	0	2.41	3.57	79.46	Calibrated

Figure S12. Modelled varicella deaths and deaths from Brisson and Edmunds [28], by age category

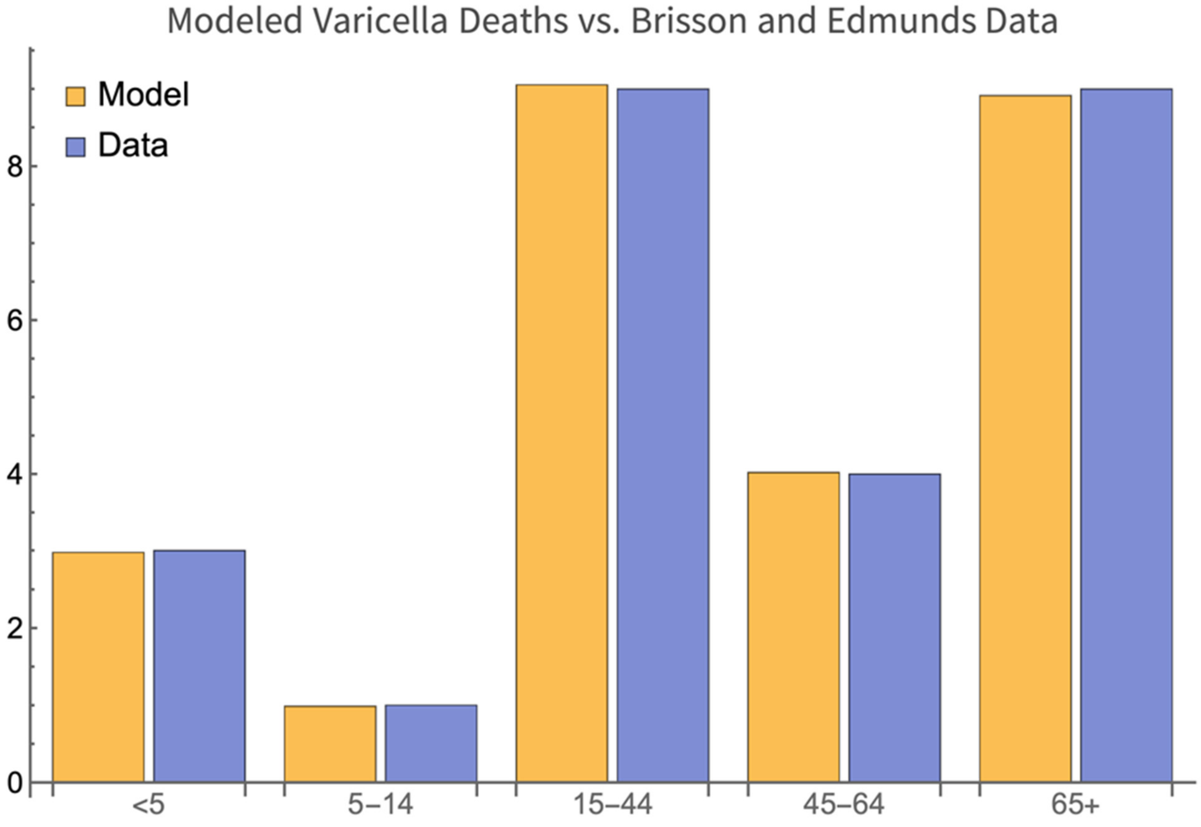

Figure S13. Modelled HZ deaths and deaths from Brisson and Edmunds [28], by age category

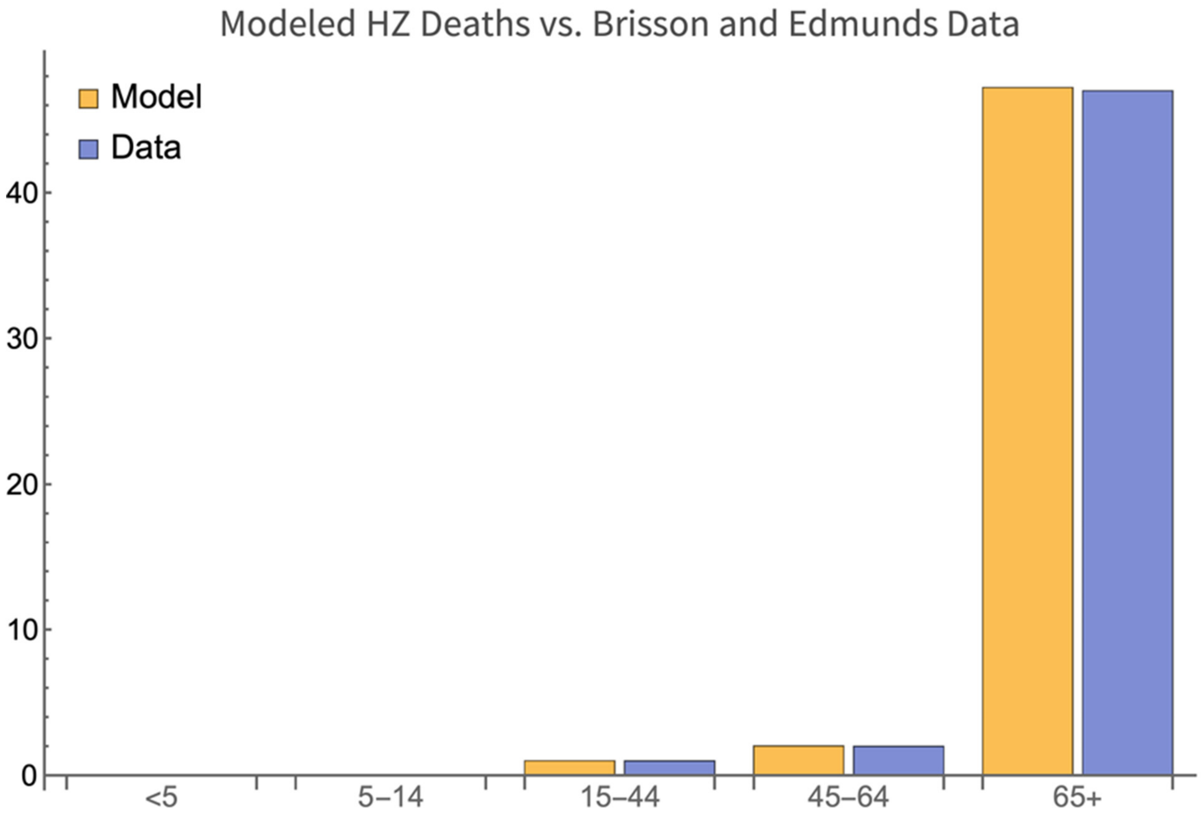

S6.4 Health Utilities and Cost

Table S10. Health utilities weights

Age group, years	Healthy [27]	Natural varicella, [28]	Breakthrough varicella [28]	Uncomplicated HZ* [29]	Postherpetic neuralgia [30]
< 15	1.000	0.791	0.919	0.870	0.657
[15,18)	1.000	0.739	0.919	0.870	0.657
[18,25)	0.923	0.662	0.842	0.793	0.580
[25,35)	0.916	0.655	0.835	0.786	0.573
[35,45)	0.892	0.631	0.811	0.762	0.549
[45,55)	0.858	0.597	0.777	0.728	0.515
[55,65)	0.820	0.559	0.739	0.690	0.477
[65,70)	0.784	0.523	0.703	0.654	0.441
[70,75)	0.784	0.523	0.703	0.628	0.441
≥ 75	0.717	0.456	0.636	0.561	0.374

* The model assumes health utilities for uncomplicated breakthrough HZ equals those of uncomplicated HZ

Table S11. Health care resource utilization unit costs [12]

Parameters	Unit costs
Varicella-related	
GP visit	£62.64
OTC drugs	£3.48
Hospitalization day	£595.07
< 15 years old	£595.07
≥ 15 years old	£425.94
Hospital treatment	£1,083.77
HZ-related	
GP visit	£94.75
Hospitalization day	
< 70 years old	£244.54
≥ 70 years old	£281.12
PHN treatment cost per case	£425.99

Table S12. Parameters for indirect costs associated with varicella and HZ

	Cases associated with workdays lost, % [4]	Workdays, if lost [4, 28]	Cost of work loss *, £ [31]
Varicella-related, by year of age group			
< 15	19.6	0.60	8.31
[15,45)	71.7	5.53	280.13
[45,65)	73.0	5.70	293.97
≥ 65	0.0	-	0.00
HZ-related, by year of age group			
< 18	0.00	-	0.00
[18,65)	50.79	10.00	358.83
≥ 65	0.00	-	0.00

* The cost per workday lost is assumed to be £70.65 [31].

S7 Sensitivity Analysis

S7.1 Deterministic Sensitivity Analysis

A deterministic sensitivity analysis (DSA) was conducted to examine the impact of uncertainty in key vaccine and cost parameters on the cost-effectiveness of the UVV strategies on the frontier when one parameter (or set of related parameters) is modified. Vaccination coverage parameters are varied by ±5 percentage points. Vaccine take and wanning is also varied. The cost of all vaccines is reduced by 30% (no upper value raising the vaccine cost is included). Direct and indirect costs are varied by ±20%.

S7.2 Posterior Distribution

Since the model calibration was performed sequentially on two sets of parameters, the internal variability of each set can be independently estimated, as well as forming a conservative joint distribution from their product.

The varicella susceptibility parameters $\mathrm{rr}[1], \mathrm{rr}[2], \mathrm{rr}[3], \mathrm{rr}[4]$, and Δ are assumed to follow a beta distribution with shape parameters α and β because they are probabilities with values between 0 and 1. Hence, let

$$\theta = \{\mathrm{rr}[1], \mathrm{rr}[2], \mathrm{rr}[3], \mathrm{rr}[4], \Delta\}.$$

Then, for each θ_i, the mean and variance of the respective beta distribution is given by

$$\mathbb{E}[\theta_i] = \frac{\alpha_i}{\alpha_i + \beta_i}$$

$$\mathbb{V}[\theta_i] = \frac{\alpha_i \beta_i}{(\alpha_i + \beta_i)^2(\alpha_i + \beta_i + 1)}$$

Here, we assumed that the mean $\mathbb{E}[\theta_i]$ is an optimized estimate, and the variance $\mathbb{V}[\theta_i]$ is estimated using the Hessian Matric of the log-likelihood objective function. Since the square root of the negative Hessian is an estimator of the

asymptotic covariance matrix and the square root of the diagonal elements of the covariance matrix are estimators for the standard errors (see ref). Hence if

$$\mathbb{E}[\theta] = \hat{\theta}$$

then,

$$\mathbb{V}[\theta] = (-H[\theta])^{-1}|_{\theta=\hat{\theta}}$$

The posterior distribution of the calibrated varicella parameters is computed as follows:

1. Compute the Hessian of the objective function (log-likelihood of the seroprevalence).
2. Take the diagonal elements of the inverse Hessian matrix to be the variance vector
3. Take the calibrated Varicella parameter values to be the mean vector
4. Set the mean and variance vectors equal to the actual mean and variance formulas for a beta distributed random variable

$$\hat{\theta} = \frac{\alpha}{\alpha + \beta}$$

$$(-H[\theta])^{-1}|_{\theta=\hat{\theta}} = \frac{\alpha\beta}{(\alpha + \beta)^2(\alpha + \beta + 1)}$$

5. Solve for the equation for α_i and β_i.

An empirical method to estimate the variability of the HZ reactivation parameters ω, ϕ, η, π. During the course of calibration, many parameter sets are encountered that can give a reasonable fit of the model to the HZ incidence data. A parameter set was deemed acceptable if the sum of squared errors was less than the corresponding component sum of squared errors of the final optimized parameter sets. The acceptable parameter sets are used to construct a kernel distribution for the calibration parameters. The marginal distributions for selected parameters are shown in Figure S14.

Figure S14: Marginal distribution of the selected parameters. For each component of the random variates, the bar chart shows the values drawn from the overall product distribution; The grey grid line represents the optimized value or the strategy base case value, depending on the selected component or the random variate vector. The dashed grey lines are the low and high values from the DSA strategy parameters.

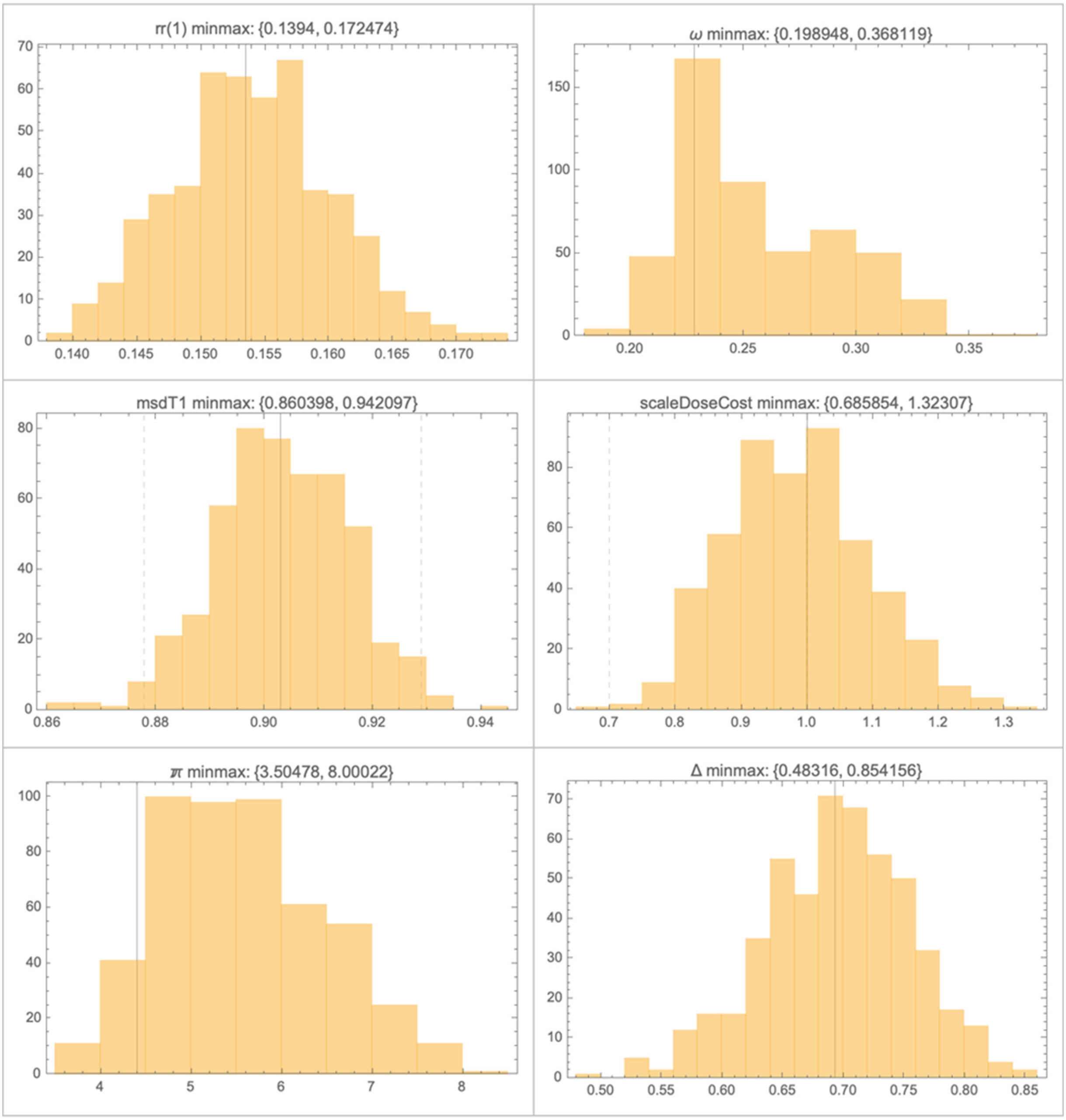

S7.3 Probabilistic Sensitivity Analysis

A joint multivariate distribution was constructed for the calibration parameters and the strategy and costs parameters were assumed to follow the beta, gamma, and log-normal distributions (Table S12). The probabilistic sensitivity analysis (PSA) used 500 random variates drawn from the joint distribution of the calibration and strategy parameters. Each strategy was simulated 500 times using these random parameter variates. The joint distributions of the resulting incremental costs and incremental QALYs gained established the probability that the UVV strategies are cost-effective compared with no UVV over a range of willingness-to-pay thresholds from the payer or the societal perspective.

Table S13. Strategy parameters and their predefined distributions used in the probabilistic sensitivity analysis

Parameter	Distribution
Coverage at 12 months	Beta [113.610, 11.2362]
Coverage at 40 months	Beta [141.912, 19.3516]
MSD first dose take	Beta [466.362, 50.0964]
GSK first dose take	Beta [443.818, 275.498]
MSD second dose take	Beta [19.6393, 8.82347]
GSK second dose take	Beta [382.854, 76.2035]
MSD waning rate per year	Gamma [9.44362, 0.0795028]
GSK waning rate per year	Gamma [50.6278, 0.0221237]
MSD dose cost	LogNormal [-0.020411, 0.103437]
GSK dose cost	LogNormal [-0.020411, 0.103437]
Varicella direct care cost	LogNormal [-0.020411, 0.103437]
Varicella indirect care cost	LogNormal [-0.020411, 0.103437]
HZ direct care cost	LogNormal [-0.020411, 0.103437]

Figure S15. Incremental cost-effectiveness plane from the 500 random variates for three strategies from the payer perspective.

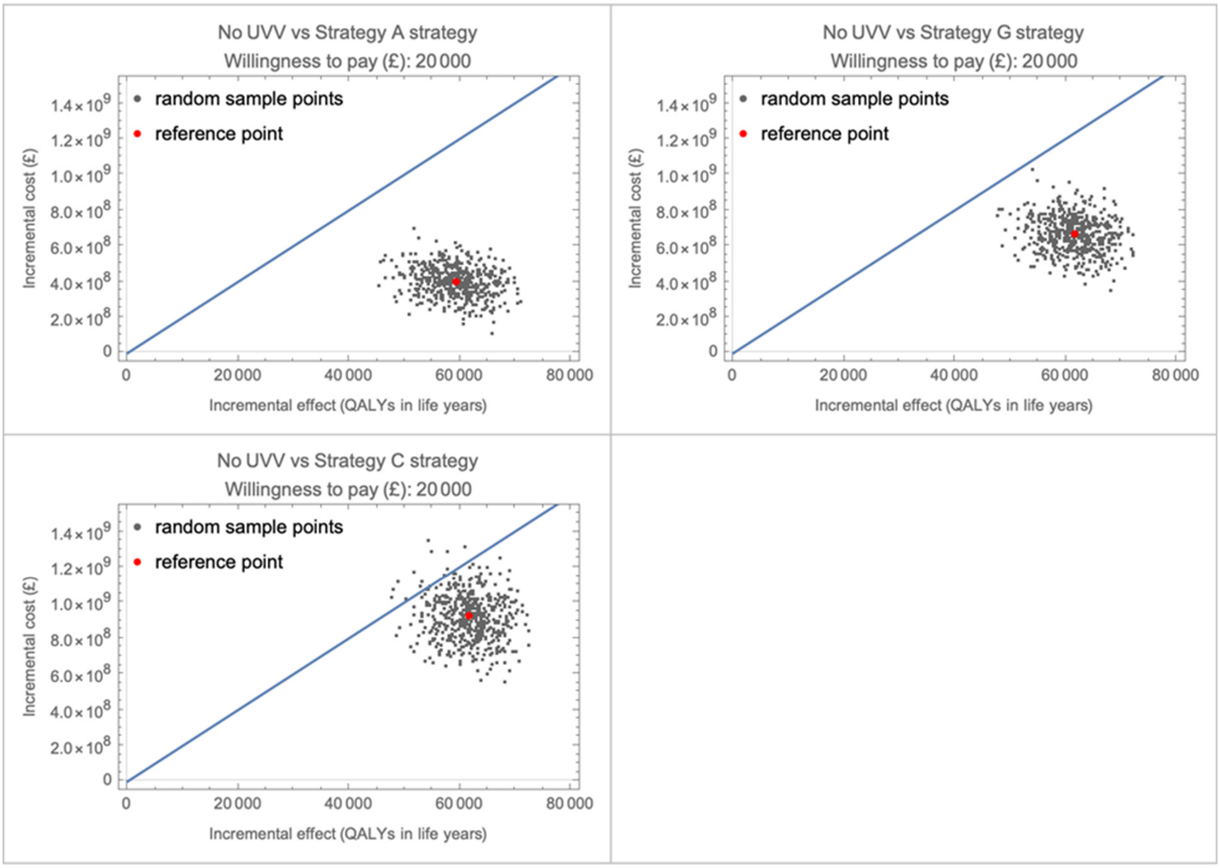

Note: The grey points represent the increment pairs generated from the randomly drawn parameter sets. The reference point is the increment pair obtained from the optimal parameter set used in the base case. The blue line passing by the origin has slope λ and shows that if the willingness to pay is above the λ value in GBP t, all the points are cost-effective strategies (i.e., all the points lie below the blue line). Each strategy is compared against the no UVV strategy.

Strategy A: MMRV-MSD (18 months); **Strategy C**: V-MSD (12 months, 18 months, catchup); **Strategy G**: V-MSD (12 months) + MMRV-MSD (40 months) + V-MSD (catchup).

Figure S16. Cost-effectiveness acceptability curve from the 500 random variates for three strategies from the payer perspective.

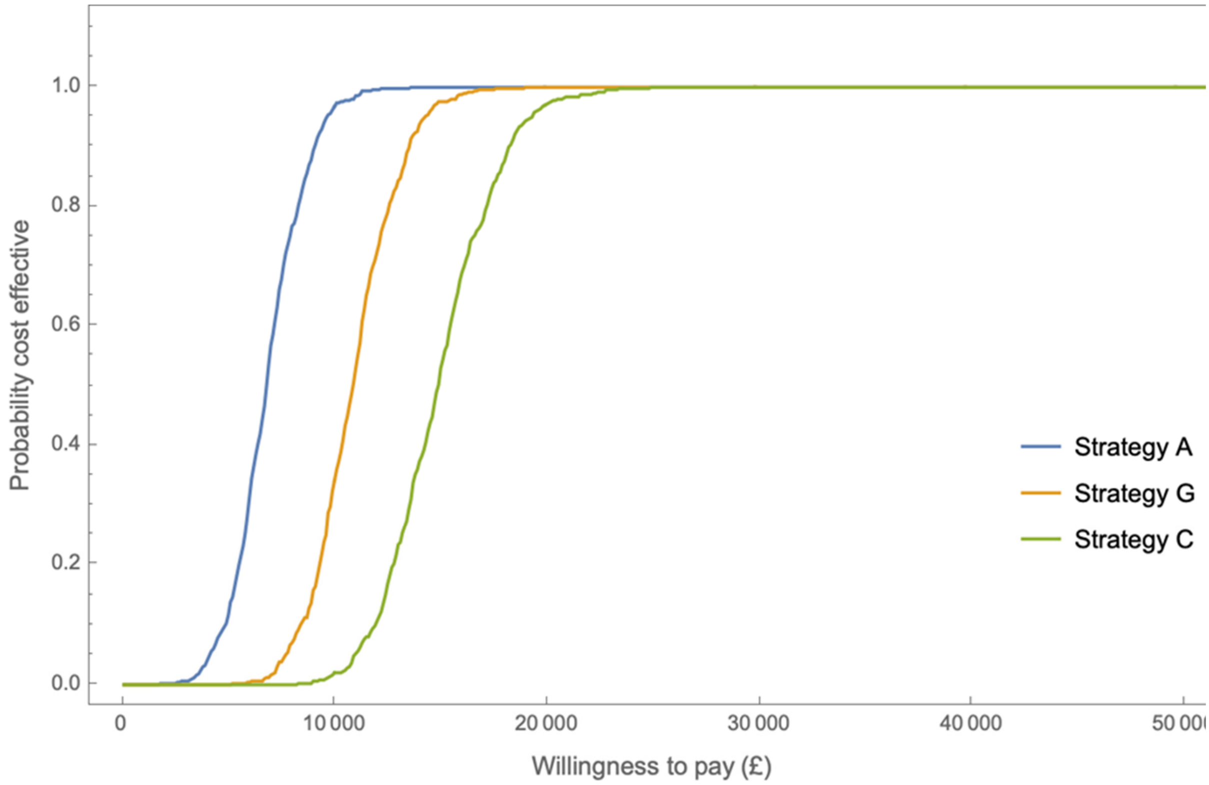

Note: Each strategy is compared against the no UVV strategy.

Strategy A: MMRV-MSD (18 months); **Strategy C**: V-MSD (12 months, 18 months, catchup); **Strategy G**: V-MSD (12 months) + MMRV-MSD (40 months) + V-MSD (catchup).

S7.4 Scenario Analysis

Table S14. HZ Vaccine properties for scenario analyses

Parameters	Age at Vaccination (years)				Source
	<71	71-75	76-80	80+	
HZ Vaccine Coverage	0%	47.9%	76.8%	0%	[32]
HZ Vaccine Take	-	55.0%	47.0%	-	[3]
Degree of Protection	-	66.9%	66.9%	-	[33]
Duration of HZ Vaccine Protection (years)	-	7.5	7.5	-	[12]

References

1. Hethcote HW. The mathematics of infectious diseases. SIAM review. 2000;42(4):599-653.

2. Brisson M, Edmunds W, Gay N. Varicella vaccination: impact of vaccine efficacy on the epidemiology of VZV. Journal of medical virology. 2003;70(S1):S31-S7.

3. Van Hoek AJ, Melegaro A, Zagheni E, Edmunds WJ, Gay N. Modelling the impact of a combined varicella and zoster vaccination programme on the epidemiology of varicella zoster virus in England. Vaccine. 2011;29(13):2411-20.

4. Akpo EIH, Cristeau O, Hunjan M, Casabona G. Epidemiological Impact and Cost-Effectiveness of Varicella Vaccination Strategies in the United Kingdom. Clinical Infectious Diseases. 2021;73(11):e3617-e26.

5. Iannelli M, Milner F. The basic approach to age-structured population dynamics. Models Methods and Numerics. 2017;10:978-94.

6. Pelovska G, Iannelli M. Numerical methods for the Lotka–McKendrick's equation. Journal of computational and applied mathematics. 2006;197(2):534-57.

7. Causes of death over 100 years [Internet]. Office for National Statistics (ons.gov.uk). Available from: https://www.ons.gov.uk/peoplepopulationandcommunity/birthsdeathsandmarriages/deaths/articles/causesofdeathover100years/2017-09-18 (accessed on 25 August 2022).

8. Births by parents' characteristics [Internet]. Office for National Statistics (ons.gov.uk). Available from: https://www.ons.gov.uk/peoplepopulationandcommunity/birthsdeathsandmarriages/livebirths/datasets/birthsbyparentscharacteristics (accessed on 25 August 2022).

9. National population projections, migration assumptions: 2018-based [Internet]. Office for National Statistics (ons.gov.uk). Available from: https://www.ons.gov.uk/peoplepopulationandcommunity/populationandmigration/populationprojections/methodologies/nationalpopulationprojectionsmigrationassumptions2018based (accessed on 25 August 2022).

10. Estimates of the population for the UK, England and Wales, Scotland and Northern Ireland - Office for National Statistics (ons.gov.uk) [Internet]. Available from: https://www.ons.gov.uk/peoplepopulationandcommunity/populationandmigration/populationestimates/datasets/populationestimatesforukenglandandwalesscotlandandnorthernireland (accessed on 25 August 2022).

11. National population projections - Office for National Statistics [Internet]. Available from: https://www.ons.gov.uk/peoplepopulationandcommunity/populationandmigration/populationprojections/bulletins/nationalpopulationprojections/2018based#toc (accessed on 25 August 2022).

12. Van Hoek AJ, Melegaro A, Gay N, Bilcke J, Edmunds WJ. The cost-effectiveness of varicella and combined varicella and herpes zoster vaccination programmes in the United Kingdom. Vaccine. 2012;30(6):1225-34.

13. Bollaerts K, Riera-Montes M, Heininger U, Hens N, Souverain A, Verstraeten T, Hartwig S. A systematic review of varicella seroprevalence in European countries before universal childhood immunization: deriving incidence from seroprevalence data. Epidemiology & Infection. 2017;145(13):2666-77.

14. Nardone A, De Ory F, Carton M, Cohen D, Van Damme P, Davidkin I, Rota MC, de Melker H, Mossong J, Slacikova M, et al. The comparative sero-epidemiology of varicella zoster virus in 11 countries in the European region. Vaccine. 2007;25(45):7866-72.

15. Wolfson LJ, Daniels VJ, Pillsbury M, Kurugöl Z, Yardimci C, Kyle J, Dinleyici EC. Cost-effectiveness analysis of universal varicella vaccination in Turkey using a dynamic transmission model. PloS one. 2019;14(8):e0220921.

16. Gauthier A, Breuer J, Carrington D, Martin M, Rémy V. Epidemiology and cost of herpes zoster and post-herpetic neuralgia in the United Kingdom. Epidemiology & Infection. 2009;137(1):38-47.

17. Fleming D, Cross K, Cobb W, Chapman R. Gender difference in the incidence of shingles. Epidemiology & Infection. 2004;132(1):1-5.

18. Edmunds W, Brisson M, Rose J. The epidemiology of herpes zoster and potential cost-effectiveness of vaccination in England and Wales. Vaccine. 2001;19(23-24):3076-90.

19. Kudesia G, Partridge S, Farrington C, Soltanpoor N. Changes in age related seroprevalence of antibody to varicella zoster virus: impact on vaccine strategy. Journal of clinical pathology. 2002;55(2):154-5.

20. Yawn BP, Yawn RA, Lydick E. Community impact of childhood varicella infections. The Journal of pediatrics. 1997;130(5):759-65.

21. Pre-primary school enrollment (kindergarten, nursery) for children aged 3-5 years from 1970 to 2016 [Internet]. StatInvestor. Available from: https://statinvestor.com/data/32099/pre-primary-school-enrollment/ (accessed on 25 August 2022).

22. Ouwens MJ, Littlewood KJ, Sauboin C, Téhard B, Denis F, Boëlle P-Y, Alain S. The impact of 2-dose routine measles, mumps, rubella, and varicella vaccination in France on the epidemiology of varicella and zoster using a dynamic model with an empirical contact matrix. Clinical therapeutics. 2015;37(4):816-29. e10.

23. Pillsbury M, Carias C, Samant S, Pawaskar M. PIN86 Modeling Performance Parametrization of Varicella Vaccines. Value in Health. 2020;23:S559.

24. Forbes H, Douglas I, Finn A, Breuer J, Bhaskaran K, Smeeth L, Packer S, Langan S.M, Mansfield KE, Marlow R, et al. Risk of herpes zoster after exposure to varicella to explore the exogenous boosting hypothesis: self controlled case series study using UK electronic healthcare data. bmj. 2020;368: I6987.

25. Walker J, Andrews N, Mathur R, Smeeth L, Thomas S. Trends in the burden of varicella in UK general practice. Epidemiology & Infection. 2017;145(13):2678-82.

26. Hobbelen PH, Stowe J, Amirthalingam G, Miller L, van Hoek A-J. The burden of hospitalisation for varicella and herpes zoster in England from 2004 to 2013. Journal of Infection. 2016;73(3):241-53.

27. Szende A, Janssen B, Cabases J. Self-reported population health: an international perspective based on EQ-5D. Dordrecht (NL): Springer. 2014.

28. Brisson M, Edmunds W. Varicella vaccination in England and Wales: cost-utility analysis. Archives of disease in childhood. 2003;88(10):862-9.

29. Pellissier JM, Brisson M, Levin MJ. Evaluation of the cost-effectiveness in the United States of a vaccine to prevent herpes zoster and postherpetic neuralgia in older adults. Vaccine. 2007;25(49):8326-37.

30. Oster G, Harding G, Dukes E, Edelsberg J, Cleary PD. Pain, medication use, and health-related quality of life in older persons with postherpetic neuralgia: results from a population-based survey. The Journal of pain. 2005;6(6):356-63.

31. Office for National Statistics. Available from: https://www.ons.gov.uk/employmentandlabourmarket/peopleinwork/earningsandworkinghours/datasets/averageweeklyearningsearn01 (accessed on 25 August 2022).

32. Public Health England. Shingles Vaccine Coverage Report (adults eligible from April to December 2020 and vaccinated to March 22021): England. Health Protection Report.15(7):1-14.

33. Tseng HF, Harpaz R, Luo Y, Hales CM, Sy LS, Tartof SY, et al. Declining effectiveness of herpes zoster vaccine in adults aged≥ 60 years. The Journal of infectious diseases. 2016;213(12):1872-5.
